# Self-Mixing Thin-Slice Solid-State Laser Metrology

**DOI:** 10.3390/s110202195

**Published:** 2011-02-15

**Authors:** Kenju Otsuka

**Affiliations:** Department of Human and Information Science, Tokai University, 1117 Kitakaname, Hiratsuka, Kanagawa 259-1207, Japan; E-Mail: ootsuka@keyaki.cc.u-tokai.ac.jp; Tel.: +81-463-58-1211; Fax: +81-463-58-9461

**Keywords:** laser-diode pumping, thin-slice solid-state laser, self-mixing modulation, optical metrology

## Abstract

This paper reviews the dynamic effect of thin-slice solid-state lasers subjected to frequency-shifted optical feedback, which led to the discovery of the self-mixing modulation effect, and its applications to quantum-noise-limited versatile laser metrology systems with extreme optical sensitivity.

## 1. Introduction

Laser feedback interferometry (LFI), also known as laser self-mixing interferometry (LSMI), is an interferometric sensing technique based on the optical mixing of the field in the laser cavity with the weak field back-reflected or back-scattered by a remote target. Herein, the historical phases leading to the development of laser feedback metrology are addressed.

### 1.1. Early Stage: Laser Feedback Interferometry with Gas Lasers

The self-mixing effect, whereby external feedback into the laser cavity induces intensity variations in the output of a gas laser, was noticed in 1960s, just after the invention of lasers [1]. A few years later, the first laser Doppler velocimeter, in which the laser cavity was used as an optical mixer, was presented [2]. It was also recognized that the fringe shift caused by an external reflector corresponds to an optical displacement of λ/2, where λ is the operating wavelength of the laser. In addition, the intensity variation corresponding to the temporal displacement of the external reflector was noticed to be comparable to conventional interferometers. According to the classification of lasers into classes A, B, and C, depending on the time scales of their dynamical variables [3], such gas lasers belong to class A, in which adiabatic eliminations of population inversion and polarization hold, and the dynamic response of the laser against the displacement is restricted merely by a photon lifetime of a laser cavity, e.g., on the order of 10^−5^. Therefore, the early-stage phenomenon should be referred to as ‘autodyne’ effect in the self-mixing laser interferometry rather than the ‘self-modulation’ originating from the dynamic effect in lasers.

### 1.2. Dynamic Effect in Self-Mixing Class-B Lasers and Versatile Optical Metrology Applications

Following the self-mixing interferometry with gas lasers, the dynamic effect was identified in self-mixing class-B lasers, in which the laser dynamics are governed by the fluorescence (carrier) lifetime of the active medium and the photon lifetime of the laser cavity.

The self-mixing modulation effect as well as nonlinear dynamics featuring chaotic behaviors in class-B lasers were initiated by Otsuka [4,5] using a thin-slice LiNdP_4_O_12_ (LNP) laser with coated end mirrors under various external perturbations including frequency-shifted or delayed optical feedback [4,5]. Later, the self-mixing effect in a CO_2_ laser was reported by Churnside [6], and the optical feedback effect in a laser diode was reported by Lang and Kobayashi [7], Shinohara *et al*. [8], Beheim and Fritschusing [9] and others. By using the laser rate equation model, it was shown that the laser is modulated due to the interference between a lasing field and a feedback field, in which the effective intensity modulation index is greatly pronounced in proportion to the fluorescence-to-photon lifetime ratio, which reaches on the order of 10^5^–10^6^ in thin-slice solid-state lasers, independently of the laser output power [4,5,10].

The feedback interferometry in class-B lasers has attracted considerable attention, mainly because class-B lasers are much more sensitive to perturbations from the outside world than conventional gas lasers [11]. The advantages of this technique with class-B lasers are sensitivity, compactness, low-cost, and simple experimental setup, *etc*. Owing to these advantages, many applications using this technique have been demonstrated, including ranging [12,13], velocimetry [8,14–25], displacement [26], vibrometry [27–29] and so on.

Lacot *et al*. performed self-mixing laser imaging of an object in turbid media [30,31] followed by 3-dimensional imaging [32] as well as cell imaging [33]. It has also been used widely in biomedical optics for blood pressure pulse registration, continuous blood pressure registration by Meigas *et al*. [34,35] and measurement of skin vibration due to MMG by Courteville *et al*. [36].

Another new application of feedback interferometry with class-B lasers is the analysis of particle size and flow velocity. Otsuka *et al*. [37–43] measured the particle size of Brownian particles as well as self-mobile planktons and flow velocity by a laser-diode-pumped self-mixing thin-slice solid-state laser. Zakian *et al*. [44,45] measured the particle size and flow velocity by a laser diode feedback interferometry (LDFI). Wang *et al*. [46] extracted the particle size distribution from the power spectrum of LDFI with the aid of an improved inverse algorithm.

In this review paper, the basic theoretical interpretation of the self-mixing modulation effect in class-B lasers and several distinct applications of laser-diode-pumped, self-mixing thin-slice solid-state lasers with extreme optical sensitivity are described.

## 2. Basic Scheme of Thin-Slice Solid-State Lasers with Laser-Diode Pumping

To begin with, let me show the basic scheme of thin-slice solid-state lasers with laser-diode pumping commonly employed in self-mixing laser metrology systems, which will be described in this review article.

**Figure 1.**
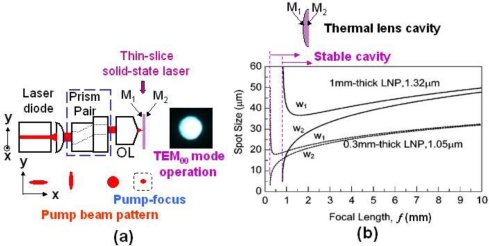
**(a)** Basic configuration of thin-slice solid-state laser with laser-diode pumping; **(b)** Spot size vs focal length of the thermal lens for LNP lasers.

The optical pumping set-up of thin-slice solid-state lasers is shown in Figure 1(a). The collimated elliptical beam from the laser diode (LD), whose lasing wavelength is tuned to the absorption wavelength of the active ion in solid-state laser (e.g., 808 nm for Nd-doped lasers), is shaped into a circular beam using a pair of anamorphic prisms and then focused on the mirror M_1_ by a microscope objective lens, in which pure symmetrical end pumping is established. The change in the pump beam profile is depicted in the figure.

The thin-slice laser consists of a platelet laser crystal with mirrors coated on end surfaces (M_1_: anti-reflection at pump wavelength and 99.9% reflection at lasing wavelength; M_2_: 99% reflection at lasing wavelength).

Due to the heat generation caused by the non-radiative process, a gradient refractive index change is formed in the crystal reflecting the parabolic temperature distribution along the radial direction. If we use laser crystals with a large absorption coefficient for the pump light, e.g., LNP, highly-doped Nd:GdVO4, and Yb:YAG, the heat is localized around M_1_. Consequently, a stable laser cavity consisting of a concave mirror (radius of curvature, *R*_1_) and a flat mirror (*R*_2_ = ∞) is formed through the thermal lens effect as depicted in the upper inset of Figure 1(b), where the focal length of a thermal lens is given by:

(1) $$f=\frac{2K}{Ql}{\left[\frac{\mathrm{dn}}{\mathrm{dT}}+\alpha (n_{0}-1)\right]}^{-1}$$

where *K* is the thermal conductivity, *Q* is the heat generated by unit volume, *dn/dT* is the coefficient of thermal refractive index change, α is the coefficient of thermal expansion, and *n*_0_ is the refractive index.

The cavity stability condition is given by −1≤*g*_1_*g*_2_ ≤1, where $g_{i}=1-\frac{l}{R_{i}}$ (*R*_i_ : radius of curvature of end mirrors), and the spot sizes at the end mirrors, *w*_1_, *w*_2_, are given by:

(2) $$w_{i}^{2}=\frac{4\pi n_{0}l}{g_{i}}\sqrt{\frac{g_{1}g_{2}}{1-g_{1}g_{2}}}$$

Example results for thin-slice LNP lasers for oscillating wavelengths of 1.05 μm and 1.32 μm are shown in Figure 1(b) assuming different crystal (*i.e.*, cavity) thickness, *l*, where spot sizes at two mirrors are shown as a function of the focal length of a thermal lens, *f =R*_1_/2 [47]. The stable region is indicated by the arrows. The stable cavity condition is found to be attained because the focal length is on the order of several millimeters under the usual pumping conditions, yielding a stable fundamental TEM_00_ operation that is realized in the simplest thin-slice laser configuration suitable for metrology applications. As for other laser materials which appear in this review paper, the cavity stability condition is established as well under the usual pumping conditions.

## 3. Self-Mixing Modulation Effect in Class-B Lasers toward Laser Doppler Metrology

### 3.1. Rate Equation Model

**Figure 2.**
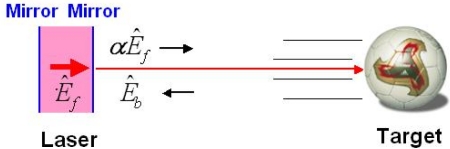
Field interference scheme in self-mixing lasers.

First, the physical interpretation is given for the self-mixing modulation effect [4,5] using Figure 2.

If we consider the interference between a lasing field and the coherent component of a feedback field from a target, the combined optical field is given by:

(3) $${\hat{E}}_{\mathrm{out}}=\alpha {\hat{E}}_{f}+{\hat{E}}_{b}$$

where *Ê_f_* is the lasing light field, *Ê_b_* is the feedback light field *α* is the field amplitude transmittance of the output mirror. As a result, the optical intensity of the combined field is expressed by:

(4) $$s={\left|{\hat{E}}_{\mathrm{out}}\right|}^{2}={\left|\alpha {\hat{E}}_{f}\right|}^{2}+{\left|{\hat{E}}_{b}\right|}^{2}+\alpha {\hat{E}}_{f}{{\hat{E}}_{b}}^{*}+\alpha {{\hat{E}}_{f}}^{*}{\hat{E}}_{b}.$$

The last two terms represent the interference effect and can be rewritten as:

(5) $$\alpha {\hat{E}}_{f}{{\hat{E}}_{b}}^{*}+\alpha {{\hat{E}}_{f}}^{*}{\hat{E}}_{b}=2\alpha \left|{\hat{E}}_{f}\right|\left|{\hat{E}}_{b}\right|\text{cos}\;\omega_{m}t.$$

where ω_m_ is the angular beat frequency between two fields.

Inserting Equation (5) into Equation (4), and setting *s_o_* = |*Ê_f_*|^2^:

(6) $$s=s_{o}(1+m\;\text{cos}\;\omega_{m}t)\alpha^{2},$$

under the weak feedback condition, *i.e*., |*Ê_b_* / *Ê_f_*|^2^ ≪ 1. Here, *m* = 2|*Ê_b_* / *αÊ_f_*| and *R_f_* ≡ |*Ê_b_* / *αÊ_f_*| is the field amplitude feedback ratio.

From the relationship between the lasing light intensity inside the cavity, *s*_o_, and the output laser intensity, *s*, in the stationary state, the intensity transmittance (*i.e.*, intensity loss, α^2^) of the output mirror is considered to be modulated at the beat frequency between the two fields, *f*_m_ = ω_m_/2π.

On the other hand, the rate equations for class-B lasers are given by:

(7) $$\begin{array}{l} \mathrm{dN}/\mathrm{dt}=W-N/\tau -B_{c}\mathrm{NS},\\ \mathrm{dS}/\mathrm{dt}=B_{c}\mathrm{NS}-S/\tau_{p}+ɛN/\tau . \end{array}$$

where *N* is the population inversion density, *S* is the photon density which is proportional to the lasing light intensity, *W* is the pumping rate, *B*_c_ is the stimulated emission rate, τ, τ_p_ are fluorescence lifetime and photon lifetime, respectively, and ε is the spontaneous emission coefficient.

Assume that the intensity loss of the output mirror (α^2^), which is inversely proportional to the photon lifetime, is modulated at the beat frequency as mentioned above, then after the standard normalizations, the following rate equations are derived:

(8) $$\begin{array}{l} \mathrm{dn}/\mathrm{dt}=w-n-\mathrm{ns},\\ \mathrm{ds}/\mathrm{dt}=\mathrm{Ks}[n-(1+m\;\text{cos}\;\omega_{m}t)]. \end{array}$$

where *w* = *W*/*W*_th_ (*W*_th_: threshold pumping rate), *n* = *N*/*N*_th_ (*N*_th_ : threshold population inversion), *s* = *S* / *S̄*(*w* = 2) (*S̄*(*w* = 2): steady-state photon density at *w* = 2), time is normalized by τ and the spontaneous emission term is neglected because ε << 1 in general. *K* = τ/τ_p_ is the lifetime ratio. It should be noted that the effective modulation index, *mK*, is enhanced by *K*-value. As for thin-slice solid-state laser, extremely large *K*-values on the order of 10^5^∼10^7^ are attained [10] because of the short photon lifetimes inherent to short cavities, whereas in laser diodes *K* = 10^3^.

### 3.2. Generalized Class-B Laser Equation with Frequency-Shifted Optical Feedback

The generalized dynamical equations for self-mixing lasers can be obtained as follows by extending the Lang-Kobayashi equations [7] to include multiple (*n*) frequency-shifted feedback lights [37]:

(9) $$dN(t)/dt=\{w-1-N(t)-[1+2N(t)]E{\left(t\right)}^{2}\}/(K/2)$$

(10) $$dE(t)/dt=N(t)E(t)+R_{f}E(t-t_{D})\Sigma \text{cos}\;\Psi_{k}(t)+{\left\{2ɛ[N(t)+1]\right\}}^{1/2}\xi (t)$$

(11) $$d\phi (t)/dt=R_{f}[E(t-t_{D})/E(t)]\Sigma \;\text{sin}\;\Psi_{k}(t)$$

(12) $$\Psi_{k}(t)=\Omega_{D,k}t-\phi (t)+\phi (t-t_{D})-(\Omega_{o}+{\Delta \Omega }_{k}/2)t_{D},k=1,2,\;\;\ldots ,n$$

where *E*(*t*) = (*g*τ)^1/2^*E*(t) is the normalized field amplitude, *N*(*t*) = *gN*_th_τ_p_(*N*(t) /*N*_th_ − 1) is the normalized excess population inversion where *N*_th_ is the threshold population inversion. *g* is the differential gain coefficient, where gain is defined as *G* = *G*_th_ + g(*N*(t) − *N*_th_). *w* = *W*/*W*_th_ is the relative pump rate normalized by the threshold, *ϕ*(*t*) is the phase of the lasing field, Ψ_k_(t) is the phase difference between the lasing and the *k*-th feedback field, *R_f_* is the common amplitude feedback coefficient for all feedback fields. Ω_D,k_ = ω_D,k_/κ is the normalized instantaneous frequency shift of the feedback light from the lasing frequency, Ω_o_ = ω_o_/κ, of the *k*-th target, ΔΩ_k_ = Δω_k_/κ is the constant frequency shift provided by acousto-optic modulator. *t* and *t_D_* are the time and the delay time normalized by the damping rate of the optical cavity *κ* = (1/2τ_p_). The last term of Equation (10) expresses the quantum (spontaneous emission) noise, where *ε* is the spontaneous emission coefficient and ξ *(t)* is the Gaussian white noise with zero mean and the value < ξ (*t*) ξ (*t’*)> = *δ*(*t − t’*) that is *δ*-correlated in time. In the short delay limit of *t*_D_ << 1 and in the case of a single feedback beam *k* = 1, Equations (9–12) are reduced to the rate equation, Equation (8). Generally speaking, by using self-mixing thin-slice solid-state lasers with extremely large *K* values versatile laser Doppler measurements can be performed with extreme optical sensitivity.

### 3.3. Comparison of Self-Mixing Thin-Slice Solid-State Laser Doppler Metrology with Conventional Measurement Systems

**Figure 3.**
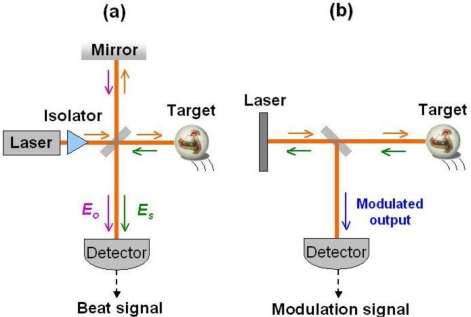
Comparison of **(a)** conventional interferometric measurement and **(b)** self-mixing laser measurement.

In the conventional interferometric laser Doppler measurement systems depicted in Figure 3(a), a reference lasing light and a scattered light from the target are combined on the photo-detector and the electric beat signal, which is proportional to the product, *Ê_o_Ê_s_*, is measured. Since the feedback light is extremely weak, in general, the highly sensitive photo-detector followed by a sophisticated signal processing system is required to extract the Doppler signal embedded in the noise.

In the self-mixing laser Doppler measurement systems illustrated in Figure 3(b), the laser acts as a quantum-noise-limited mixer-oscillator driven by a Doppler beat optical wave and the photo-detector is employed just to monitor the laser output intensity. Moreover, the optical sensitivity is determined only by the feedback ratio *R_f_* and enhanced by *K*-value, independently of the laser output power. In addition, the system is simple and self-aligned, with no solid interferometer being required.

## 4. Multiple-Channel Self-Mixing Laser Doppler Measurement with Carrier-Frequency-Division Multiplexing

As we discussed in the previous Section 3, thin-slice solid-state lasers having large lifetime ratios *K*, ensure high optical sensitivity and enable us to perform real-time nanometer-scale vibration measurements under the intensity feedback ratio below −100 dB [28,29]. Compact, inexpensive real-time laser-Doppler metrology systems could be achieved, if we could make simultaneous measurements of multiple targets or different positions of the target using one laser utilizing the high optical sensitivity inherent to thin-slice self-mixing solid-state lasers. This section describes such simultaneous real-time measurements of targets with one thin-slice solid-state laser, multiple sets of optical frequency shifters for carrier-frequency multiplexing and a multi-channel FM-demodulation circuit [37,41].

### 4.1. Multi-Channel Real-Time Vibration Measurements

#### 4.1.1. Experimental Results of 2-Channel Vibrometry

**Figure 4.**
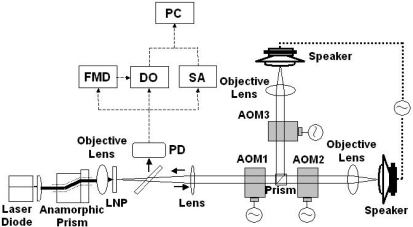
Experimental setup of two-channel self-mixing laser-Doppler measurements. FMD: 2-channel FM-wave demodulation circuit, PC: personal computer (reprinted with permission from [35]; © 2005, Optical Society of America).

The experimental setup is shown in Figure 4, where a 0.3-mm-thick LNP laser with coated end mirrors (M_1_: 95% transmittance at 808 nm, 99.8% reflectance at the lasing wavelength of 1,048 nm; M_2_: 98% reflectance at 1,048 nm) was used.

The LNP lasing wavelength λ was 1,048 nm. The threshold pump power was 30 mW and the slope efficiency was 40%. A part of the output beam (96%) was divided into two access beams and each beam was focused on a speaker through two sets of PbMoO_4_ acousto-optic modulators (AOMs), *i.e.*, optical frequency shifters. A microscope objective lens (numerical aperture: NA = 0.25) was used to focus the beams. Here, the first AOM1 introduced the common upward frequency shift of 80 MHz for two channels. The second AOM2 and third AOM3 induced −78.65 and −78.25 MHz downward frequency shift, respectively. In short, the round-trip carrier-frequency shift, 2*f*_AOM_, for two channels was *f*_c,1_ = 2.7 MHz for channel 1 and *f*_c,2_ = 3.5 MHz for channel 2, respectively. Another part (4%) of the output beam was delivered to an InGaAs photo-diode receiver (PD). The electrical signal from the photo-diode was used to measure waveforms and power spectra of modulated and demodulated outputs with a digital oscilloscope (DO) and a rf spectrum analyzer (SA).

**Figure 5.**
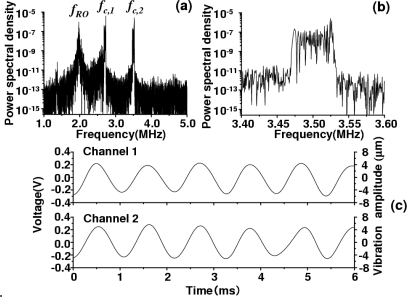
Example power spectrum of the modulated signal and demodulated output voltages. Modulation frequency *f_m·_* = 914 Hz. **(a)** Power spectrum of modulated signal; **(b)** magnified view around *f_c,2_* = 3.5 MHz; and **(c)** demodulated voltages of two channels (reprinted with permission from [35]; © 2005, Optical Society of America).

The modulated signal was composed of two FM-type intensity modulated waves whose carrier frequencies are *f*_c,1_ = 2.7 MHz and *f*_c,2_ = 3.5 MHz, respectively. As a result, the observed power spectrum consists of two power spectra around 2.7 and 3.5 MHz whose frequency components show FM sidebands. An example power spectrum of the modulated signal and a magnified view around *f*_c,2_ = 3.5 MHz are shown in Figures 5(a) and 5(b) respectively, where the common sinusoidal voltage was applied to the two speakers at modulation frequency *f*_m_ = 914 Hz. The intrinsic relaxation oscillation intensity noise is indicated by *f*_RO_ in the figure. The effect of relaxation oscillations on vibration measurements was not observed.

Figure 5(c) shows demodulated output voltages *V*_o_(t) of two channels corresponding to Figures 5(a), each of which corresponds to a velocity variation *v*(t) of each speaker’s rough surface. The vibration waveform, *D*_p_(t), can be deduced by integrating *v*(t) over time. In the present case, the vibration waveform *D*_p_(t) is similar in shape to *v*(t), *i.e.*, *V*_o_(t). Due to the poor quality of the speakers, slightly irregular amplitude variations are seen. It was confirmed that the cross-talk between two speakers was absent. In short, the demodulated output waveform of one speaker was kept unchanged when we blocked an access beam to another speaker. This is because that the nonlinear wave mixing between the strong lasing field at frequency, *f*, and extremely weak re-injected fields in the laser, *f* + *f*_c,1_ and *f* + *f*_c,2_, e.g., combination-tone polarizations are not created near re-injected light frequencies under such weak feedback conditions, especially when two carrier frequencies are incommensurate like the present frequency arrangement.

**Figure 6.**
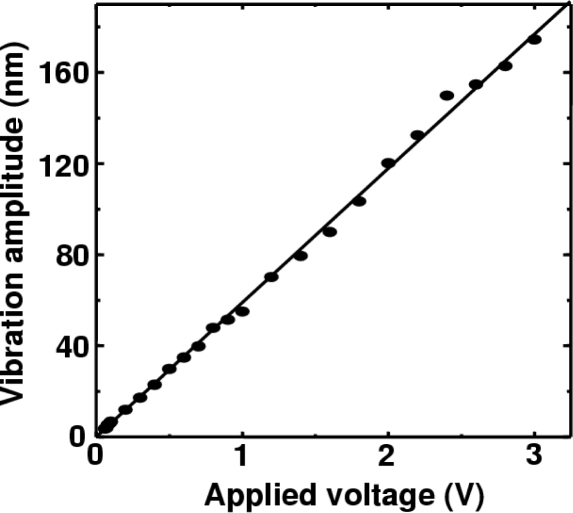
Vibration amplitude versus voltage applied to the speaker. Modulation frequency *f*_m_ = 8 kHz (reprinted with permission from [35]; © 2005, Optical Society of America).

A super-heterodyne method with a central frequency of 10.7 MHz was employed to demodulate the FM wave; frequencies of external oscillators were tuned to provide the 10.7 MHz central frequency to both channels and the amplifier in each channel had a maximum gain of 20 dB and a 3-dB bandwidth of 111 kHz. In Figure 5(c), the displacement *D*_p_(t) of each speaker’s surface is also shown on the right vertical axis, where the displacement is related to the output voltage as *D*_p_/*V*_o_ = 20 [μm/V]. To obtain such a voltage-displacement relationship, we used Hilbert transformation of the modulated output waveform and phase-sensitive detection by using LabView software on a personal computer (PC), assuming the relation *D*_p_ (t) = λ ΔΦ(t)/2π, where ΔΦ(t) is the analytic phase difference between the carrier (reference) wave and the modulated wave, as calculated from Gabor’s analytic signal [28,29]. The analytic phase Φ_A_ is related to analytic signal *V*_A_ and its time average <*V*_A_> by *V*_A_ − <*V*_A_> = *R*_A_(t)*exp*[*i*Φ_A_ (t)]. Here, *V*_A_(t) = *I*(t) + *iI*_H_(t), where *I*(t) is the time series of the scalar intensity and *I*_H_ is its Hilbert transform. The maximum vibration amplitude *V*_max_ was also evaluated accurately from the FM modulation index β = 32 determined from ratios of FM sideband intensities to the carrier intensity of modulated signals using the relation *V*_max_ = λβ/2π yielding *D*_p_ = 5.3 μm, which parallels the vibration amplitude shown in Figure 5(c) [right vertical axis] calibrated from the phase-sensitive detection on the PC. From the systematic measurement for different voltages to the speaker, the vibration amplitude was found to vary in the relation of *V*_max_ = 60 [nm/V] at *f*_m_ = 8 kHz as shown in Figure 6. Simultaneous real-time measurements were performed successfully even if the speakers were driven at different frequencies.

#### 4.1.2. Numerical Result

**Figure 7.**
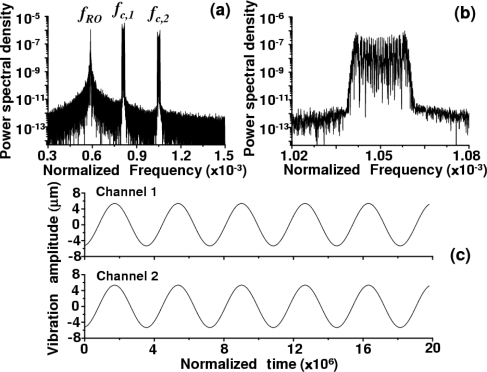
Numerical results for two-channel operations. **(a)** Power spectrum of modulated signal, **(b)** magnified view around *f_c,2_* = 3.5 MHz, and **(c)** vibration waveforms. Parameters are given in the text (reprinted with permission from [35]; © 2005, Optical Society of America).

To confirm the idea of carrier-frequency-multiplexed self-mixing laser-Doppler vibrometry using one laser and reproduce the experimental results, the numerical simulation of the model equation of a laser subjected to frequency-modulated multiple feedback lights Equations (9–12), assuming:

(13) $$\Omega_{D,k}=\Delta \Omega_{k}+\beta \text{sin}\Omega_{m,k}t,$$

where Ω*_m,k_* = ω*_m,k_*/κ is the normalized angular frequency of the applied voltage to the *k*-th target. The numerical results are shown in Figure 7 for the case of two feedback fields as demonstrated in the experiment. The photon lifetime τ_p_ of the present laser was estimated from the pump-dependent relaxation oscillation frequency, *f*_RO_, using the relation *f*_RO_ = (1/2π)[(*w* − 1)/ττ_p_], where *w* = *P*/*P*_th_ is the relative pump power and τ = 120 μs is the LNP’s fluorescence lifetime. The estimated value was τ_p_ = 300 ps, yielding a lifetime ratio of *K* = 2 × 10^5^. Other adopted parameter values, which are relevant to the present experiment, are *w* = 6.45, carrier frequencies Δ*f_1_* = *f_c,1_* = 2.7 MHz, Δ*f_2_* = *f_c,2_* = 3.5 MHz, and modulation frequencies *f*_m,1_ = *f*_m,2_ = 914 Hz, β = 32, delay time = 6 ns, amplitude feedback coefficient *R_f_* = 10^−5^ (*i.e.*, intensity feedback ratio of −100 dB) and *ε* = 10^–11^.

A sample calculated power spectrum of the modulated signals and a magnified view around *f*_c,2_ = 3.5 MHz are shown in Figures 7(a) and 7(b), respectively, where the common sinusoidal voltage was applied to the two speakers. Vibration waveforms for two channels, which were calculated from the modulated waveform by the phase-sensitive detection using Gabor’s analytic phases on PC, are shown Figure 7(c). The numerical results well reproduce the simultaneous independent measurement of vibrations of two targets shown in Figure 5. The range of acceptable levels of intensity feedback ratio, 10 log *R_f_*^2^, in the present system was estimated to be −100 dB from the correspondence between results of experiment and simulation.

#### 4.1.3. 3-Channel Real-Time Measurements of Nanometer Vibrations

A variety of three-dimensional (3-D) metrology systems have been developed for measuring moving objects and turbulent fluid flow [48–55]. Typical examples include 3-D laser-Doppler vibrometry systems using three oblique incident beams impinging on the target and particle image velocimetry systems. In these state-of-the-art 3-D optical metrology systems reported so far, beat signals or interference fringes between the lasing output field and the scattered optical fields from moving targets are detected using sophisticated optics and highly sensitive electronics.

On the other hand, the simplest, self-aligned 3-D nanometer-scale metrology systems with extreme optical sensitivity, without using any sophisticated optical interferometers or highly sensitive electronics, can be realized with a 3-channel self-mixing thin-slice solid-state laser metrology system.

**Figure 8.**
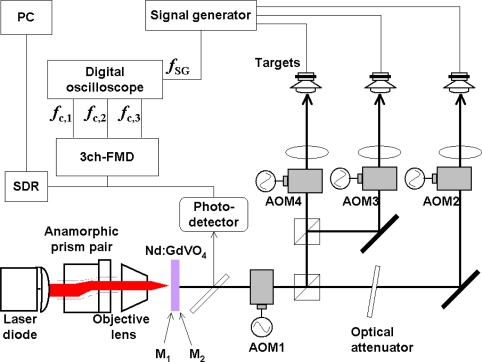
Experimental setup of three-channel self-mixing laser-Doppler measurement system (reprinted with permission from [41]; © 2009, Optical Society of America).

Experimental results of simultaneous measurements of three different vibrating targets are shown. The experimental setup is shown in Figure 8, where a 3 at.% Nd-doped 1-mm-thick Nd:GdVO_4_ laser with coated end mirrors (M_1_: 95% transmittance at 808 nm, 99.8% reflectance at the lasing wavelength of 1,064 nm; M_2_: 98% reflectance at 1,064 nm) and three sets of TeO_2_ acousto-optic modulators (AOMs) are used.

The photon lifetime τ_p_ of the present laser was estimated to be 235 ps from the pump-dependent relaxation oscillation frequency, *f*_RO_, yielding a lifetime ratio of *K* = 4.05 × 10^5^, assuming τ = 90 μs. Here, AOM1 introduced a common upward frequency shift of 79.85 MHz for all three channels. AOM2, 3 and 4 provided 78.85-, 78.1-, and 77.6-MHz downward frequency shifts, respectively. In short, the round-trip carrier-frequency shifts 2*f*_AOM_ for three channels were *f*_c,1_ = 2 MHz for channel-1, *f*_c,2_ = 3.5 MHz for channel-2, and *f*_c,3_ = 4.5 MHz for channel-3. The electrical signal from the photodiode was delivered to a software defined radio (RFSPACE Inc. SDR-14: DC-30 MHz) or three-channel frequency-modulated wave demodulation circuit (analogue FM-demodulator) having three different outputs matched to carrier frequencies *f*_c,1_, *f*_c,2_, and *f*_c,3_, depending on the purpose.

**Figure 9.**
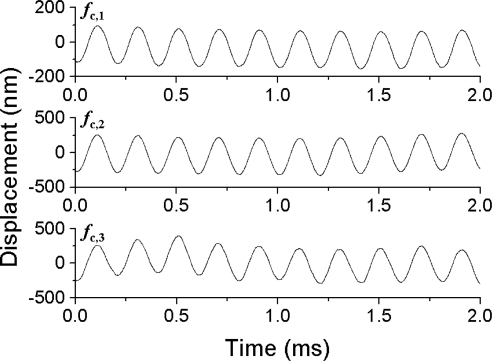
Example vibration waveforms of three piezoelectric elements measured by the three-channel analog FM demodulation circuit. Modulation frequency f_m_ = 5 kHz. Applied voltage V_a_ = 10 V_pp_ (reprinted with permission from [41]; © 2009, Optical Society of America).

Here, three vibrating targets, each of which consisted of a mirror attached to a piezoelectric-element, were used as shown in Figure 8. Figure 9 shows vibrating waveforms calculated by the integral of the output signals *V*_o_(t) from the FM-demodulator which is proportional to the instantaneous velocity of the piezoelectric-element over time for each of the three channels. Here, the displacement, *D*_p_(t) = λΔΦ/2π was estimated from the Hilbert transformation of a modulated signal and the phase-sensitive detection for each channel (*i.e.*, carrier frequency) by using LabView software on a personal computer, as mentioned in 4.1.1. The measured vibration amplitude was confirmed to coincide with those measured with a capacitive displacement sensor [39]. The cross talk among three channels was completely eliminated by tuning the filter bandwidth of the FM-demodulator circuit around the carrier frequency.

**Figure 10.**
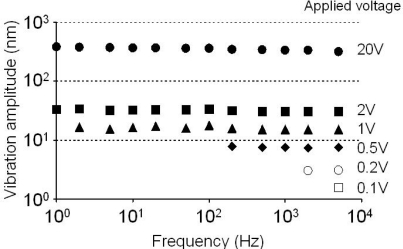
Vibration amplitude versus driving frequencies for different voltages applied to piezoelectric elements (reprinted with permission from [41]; © 2009, Optical Society of America).

The vibration amplitudes measured at different driving frequencies are summarized in Figure 10. The software-defined radio enabled us to perform phase-sensitive detection of modulated signals to obtain vibration amplitudes directly in the form of *D*_p_(t) = λΔΦ/2π. The vibration amplitude was found to be independent of the driving frequencies in the range from 1 Hz to 5 kHz for vibration amplitudes larger than 30 nm. The measurable vibration amplitude in the present three-channel real-time vibrometry system was about 2 nm at 5 kHz, as indicated by open circles in Figure 10.

The present set-up can be applied to dynamic bending measurements of metal plates by impinging access beams on at least three positions of the target. If we focus three access beams on the same position of the target from three different directions, vibration test and 3-dimensional vibration analysis of small structures, housing and components can be performed.

### 4.2. 3-Dimensional Measurement of Liquid Flow

#### 4.2.1. Dilute Sample-Flow through a Vertical Glass Column

Sensitive and rapid detection of small particles in a fluid flow is needed for many applications, such as the production of pharmaceuticals and semiconductor equipment, conservation of water quality, and marine science. For example, integrated circuits use electrical lines as narrow as nearly 0.1 μm, and highly efficient detection of micro-particles and nanometer-sized particles in washing water is required to evaluate semiconductor wafer contamination. The detection of small particles in fluid flow has been performed extensively using a magnetic flowmeter [56,57], ultrasound Doppler velocimeter [58,59], and laser Doppler velocimeter (LDV) [60–64]. The advantage of the LDV for a dilute sample-flow compared with conventional measuring techniques lies in the high spatial and temporal resolution and the possibility of non-contact measurement for the detection of particles of 1 nm to 10 μm in size in a dilute sample. In the LDV, the velocity of moving particles is related to the Doppler shift frequency *f*_D_ of scattered light by [65]:

(14) $$f_{D}=\frac{{2v}_{z}}{\lambda }=\frac{2v\text{sin}\;\theta }{\lambda }.$$

Here, *λ* is the wavelength, *v* is the velocity of the moving particle, *v_z_* is its velocity along the probe beam axis, and *θ* is the angle between the velocity vector and the probe beam axis. The LDV system, which utilizes the self-mixing modulation effect in semiconductor lasers, has been applied to vibrometry [66–68], speckle pattern interferometry [69], and measurements of the “average” velocity and flow of small particles and red-blood cells [16,17,21–23,70,71].

Before presenting 3-D measurement of liquid flow, let us show the liquid flow in a vertical glass column measured with a single access beam using the same 3 at.% Nd-doped Nd:GdVO_4_ self-mixing laser metrology system as that used in the experiment described in 4.1.3 under the single-channel configuration. The distinct feature of self-mixing thin-slice solid-state laser scheme, which is not expected in self-mixing semiconductor lasers, includes the inherent high optical sensitivity due to 2–3 orders of magnitude larger *K* values, much narrower line-width as well as smaller spontaneous emission noise than LDs.

**Figure 11.**
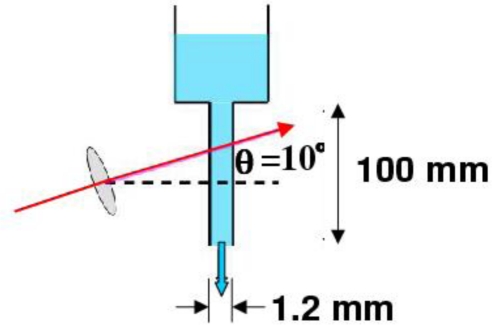
Configuration of the flow passage for a dilute sample-flow (reprinted with permission from [40]; © Copyright 2007, Optical Society of America).

The flow passage is illustrated in Figure 11. A Doppler-shift frequency can be increased with increasing the angle. Therefore, the large angle is suitable for measuring velocity distributions of liquids with high viscosities. For measuring liquid flow with lower viscosities, on the other hand, we need a narrower frequency span (*i.e.*, high frequency resolution) for power spectrum analysis to extract the information in the vicinity of 2*f*_AOM_. Therefore, the angle was set to 10° to measure liquids having various viscosities with reasonable resolutions under the same scattering scheme. In the scattering cell, 3.7 cm^3^ of fluid flowed from the reservoir at the top down through a vertical glass column of 100 mm long and 1.2 mm in diameter. Water-glycerol mixtures with various glycerol concentrations were used as liquid, and polystyrene latex standards (PS) with a diameter of 262 nm were added as the tracer. Here, the flow velocity becomes slower by adding glycerol, because the viscosity of the glycerol is much higher than that of the water. The concentration of PS particles used in the first experiment was 0.05 wt% in all mixtures.

**Figure 12.**
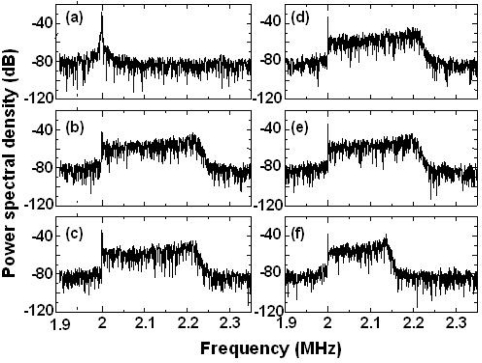
Time dependency of the power spectrum for 262-nm diameter PS particles in water-flow with concentration of 0.05 wt% **(a)** before the flow of the dilute sample and at **(b)** 0 s; **(c)** 2 s; **(d)** 5 s; **(e)** 8 s; and **(f)** 11 s. (4.799 Mbytes) (reprinted with permission from [40]; © 2007, Optical Society of America).

Figure 12 shows time-dependent power spectra of the modulated wave for PS particles in water. Before the flow, the power spectrum showed a Lorentz shape representing the Brownian motion of PS particles as will be shown in Section 5. When the dilute sample began to flow in the passage, a power spectrum with an asymmetric shape appeared on the higher frequency side with respect to the carrier frequency 2*f*_AOM_ = 2 MHz for θ > 0 (see the corresponding Supplementary Material).

**Figure 13.**
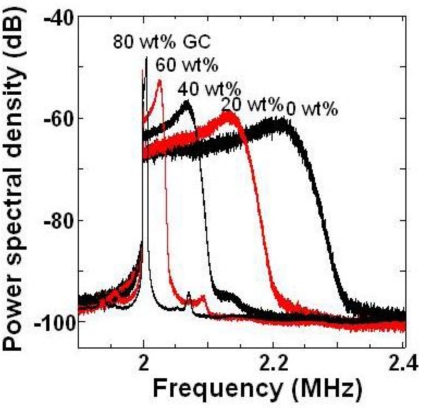
Power spectra for 262 nm diameter PS particles in various water-glycerol mixtures (reprinted with permission from [40]; © 2007, Optical Society of America).

The averaged power spectra for PS particles in various glycerol-water mixtures are shown in Figure 13.

The power spectrum became narrow and the peak frequency approached 2*f*_AOM_ as the glycerol concentration increased. This approach toward 2*f*_AOM_ resulted from a decrease in the velocity of PS particles moving in the vertical direction in the fluid flow as the glycerol concentration was increased. In other words, the velocity of the dilute sample-flow containing PS particles decreased as the viscosity of the water-glycerol mixture increased, and the decrease in velocity of PS particles led to a decrease in the Doppler frequency-shift of the modulated wave, as represented by Equation (14). The narrowing of the power spectrum with decreasing velocity of dilute sample-flow was predicted in Reference [72].

#### 4.2.2. Analysis of Experimental Results

To explain the observed power spectra, let us characterize the measured power spectra during the time period when stationary flow (*i.e.*, constant peak frequency shift) was established, as shown in Figure 12(b–e). The results of the dilute sample-flow within the pipe, the fluid flow can be treated as a laminar flow and the velocity distribution of fluid flow can be expressed using Poiseuille’s law. The Poiseuille equation gives the velocity distribution of the fluid flow as:

(15) $$v(r)=\frac{\Delta P}{4\pi \eta }(a^{2}-r^{2}).$$

where *ΔP* is the pressure difference between the two ends of the passage, *η* is the kinetic viscosity, *a* is the radius of the passage, and *r* is the distance from the center of the passage.

Let us calculate the power spectrum of light scattered from PS particles in a dilute sample-flow in the passage. The volumetric flow rate *Q*/*t* for a dilute sample-flow in the passage in the vertical direction is related to the maximum of the velocity distribution *v_max_* by:

(16) $$\frac{Q}{t}={\pi a}^{2}v_{\mathrm{avg}}=\frac{1}{2}{\pi a}^{2}v_{\text{max}}.$$

**Figure 14.**
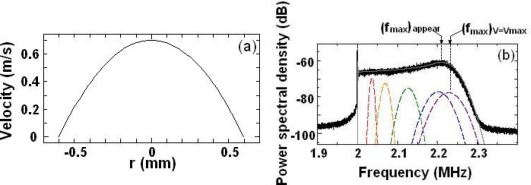
**(a)** Dependence of the calculated flow velocity in the passage on distance from the center of the passage. **(b)** Power spectrum for PS in water-flow in the passage. The black line indicates the observed power spectrum. The dashed lines indicate the power spectrum for PS particles moving with each velocity element. red: *r* = 5.5 mm, orange: 5.0 mm, green: 4.0 mm, blue: 2.0 mm, violet: 0 mm. The thin gray curve indicates the summation of the power spectra for all velocity elements (reprinted with permission from [40]; © 2007, Optical Society of America).

Here, *v_avg_* is the average of the velocity distribution, where *v_avg_* = (1/2)*v_max_*. The velocity distribution calculated for PS in water is shown in Figure 14(a). The theoretical study indicates that the power spectrum of light scattered from a rotating ground glass or a particle moving uniformly is a Gaussian spectrum whose width depends on the transient time of a single irregularity or a particle across the incident light beam [72]. Therefore, the observed power spectrum for PS particles in dilute sample-flow can be interpreted as the summation of Gaussian spectra with continuously changing width and peak frequency as:

(17) $$I(\omega )=\sum_{i}\frac{A_{i}}{w_{i}}\text{exp}\left\{-\frac{{\left[\omega -2\pi (2f_{\mathrm{AOM}}+f_{D,i})\right]}^{2}}{w_{i}^{2}}\right\}.$$

where subscript *i* is an element of the velocity in the passage. Empirical parameters *A* and *w* are the amplitude and width of the Gaussian spectrum relating to the velocity of the particle, respectively. *f_D_* is the Doppler shift frequency of scattered light given by Equation (14). Figure 14(b) shows the calculated power spectrum for each PS particle moving in a dilute sample-flow together with the experimental power spectrum.

**Figure 15.**
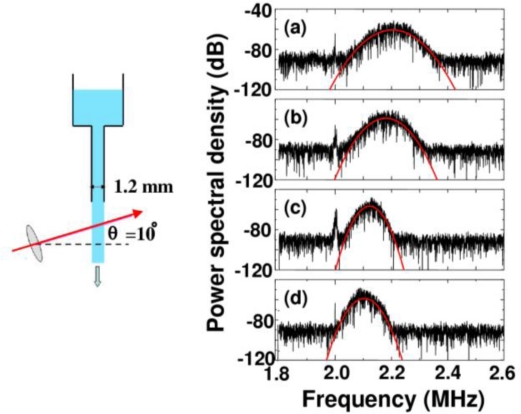
Time dependency of the power spectrum for 262-nm-diameter PS particles in dropped water with 0.05 wt% concentration **(a)** 2 s, **(b)** 5 s, **(c)** 8 s, **(d)** 11s. The red lines indicate the results of curve fitting using a Gaussian function. See the Supplementary Material (1.763 M bytes) (reprinted with permission from [40]; © 2007, Optical Society of America).

Here, the fitting procedure is as follows:

1. Thirty Gaussian functions are set within the observed power spectrum, whose center frequencies *f*_Di_ are equally spaced, to describe the velocity distributions.
2. *v*_i_-values are derived using Equation (14) and *w*_i_-values are determined using the empirical relation, *w*_i_ = 574,100*v*_i_, which was experimentally obtained from the Gaussian curve fitting of time dependent power spectra for 262-nm-diameter PS particles in ‘dropped water’ from the vertical pipe shown in Figure 15 [40]. Here, the velocity distribution of the PS particles in the light path is very small, *i.e.*, *v*(r) = *v* (constant) when the focusing point is larger than 2.5 mm below the exit of the pipe.
3. A variety of *A*_i_-values are introduced as fitting parameters for the summation of Gaussian spectra. The Gaussian spectrum broad is broadened and the peak frequency, *f*_max_ (=2*f*_AOM_ + *f*_D_), is shifted to a higher frequency range for each element with increasing *r*.

The thin grey curve indicates the sum of the Gaussian spectra for all elements. The calculated power spectrum is in good agreement with the power spectrum observed for a dilute sample-flow.

#### 4.2.3. Evaluations of Flow Velocities and Kinetic Viscositie*s*

From the excellent correspondence between the experimental power spectra and the theoretical velocity distributions discussed so far, it is concluded that the present self-mixing laser can measure velocity distributions of PS particles in a dilute sample-flow in a passage that obeys Poiseuille’s law.

Now, let us evaluate the maximum flow velocities and kinetic viscosities from experimental power spectra. The peak frequency of the observed power spectrum, *(f_max_)_appear_*, does not agree with the peak frequency of the power spectrum for a particle moving with maximum velocity at the center of the passage, *(f_max_)_V=Vmax_*, as shown in Figure 13(b). The relationship between these two frequencies was investigated analytically for dilute sample-flows with various velocities to obtain the flow velocity quickly and directly from the observed power spectrum. It was found that *(f_max_)_appear_* is proportional to *(f_max_)_V_ = _Vmax_* as *(f_max_)_V_ = _Vmax_* = 1.0783 × *(f_max_)_appear_* − 156518.

**Figure 16.**
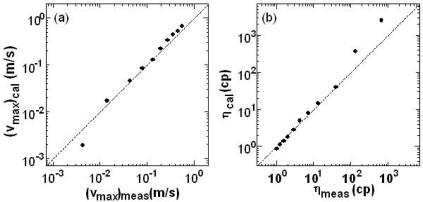
**(a)** Relationships between the maximum velocities calculated from the volumetric flow rate and obtained from observed power spectra. **(b)** Relationships between the kinetic viscosities for glycerol-water mixtures measured using an Ubbelohde capillary viscosimeter, η_cal_, and those obtained from observed power spectra, η_meas_ (reprinted with permission from [40]; © 2007, Optical Society of America).

Figure 16(a) shows the relationships between *(v_max_)_meas_* against *(v_max_)_cal_* for PS particles in glycerol-water mixtures. Here, *(v_max_)_cal_* was calculated from *(f_max_)_V_ = _Vmax_* and *(v_max_)_meas_* was obtained from the measured volumetric flow rate.

There is good agreement between *(v_max_)_cal_* and *(v_max_)_meas_*. The kinetic viscosity of the dilute sample was calculated for PS in glycerol-water mixtures from Equation (15) and the result is shown in Figure 16(b). The deviation from the calculated values in the low velocity (*i.e.*, high viscosity) region is considered to arise from the substantial contribution of Brownian motions of particles. Another systematic determination of the maximum flow velocity along the central axis will be discussed in Section 4.2.5. As shown in this section, the flow velocity as well as the kinetic viscosity for a dilute sample-flow in the passage are measured quickly and directly from the power spectrum of a modulated self-mixing laser intensity.

#### 4.2.4. Detection of Small Particles in Fluid Flow

Applying the liquid flow measurement, we can detect an extremely small amount of small particles in a fluid flow. Let us examine the concentrations that can be measured with the present self-mixing LDV system for the dilute sample-flow. In the experiment, the flow passage was set such that the sample-flow occurred through a horizontal column of a 1.2-mm diameter pipe. In this flow scheme, the flow velocity in the passage in the horizontal direction is smaller than that in the vertical direction and the time taken for the dilute sample to pass through a unit volume is long. As a result, we can measure the average power spectrum for a long period of time.

**Figure 17.**
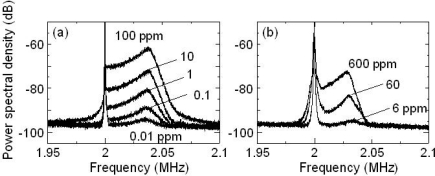
Power spectrum for **(a)** 262-nm diameter PS particles and **(b)** red blood cells in a water flow with various concentrations (reprinted with permission from [40]; © 2007, Optical Society of America).

Figure 17(a) shows average power spectra for 262-nm diameter PS in water as the concentration was decreased. The amplitude of the power spectrum decreased monotonically with decreasing PS concentration, while the power spectral profiles were unchanged.

Here, 262-nm PS particles could be detected at 0.01 parts per million (ppm) for a dilute sample-flow. An example result for dilute samples including another size particle, *i.e.*, red blood cells of sheep with a diameter of 3 μm in water, is shown in Figure 17(b). The red blood cells in the dilute sample-flow were detected at a concentration of 6 ppm.

Note that power spectral profiles for red blood cells were different from those for 262-nm PS, in which the effect of the Brownian motion of particles near the wall was different for larger particles. The measurable low concentration limit depended on a particle size since the intensity of scattered light depended on the particle size. According to the investigation of Brownian particles, which will be discussed in chapter 5, the lower bound of the concentration detected by the present self-mixing flowmetry is estimated < 1 ppm for particles of 40–500 nm diameters.

#### 4.2.5. 3-D Flowmetry

**Figure 18.**
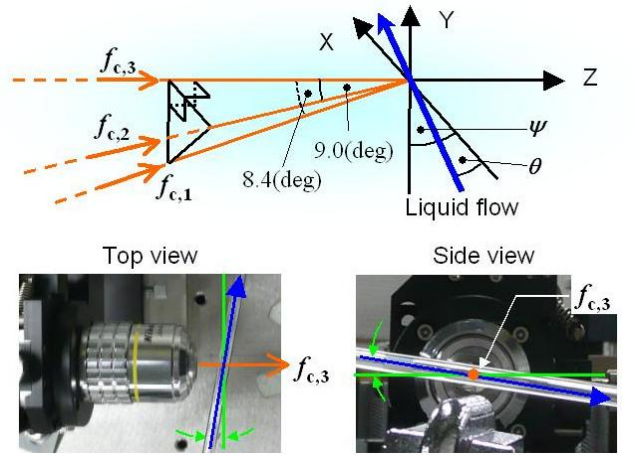
Experimental setup of 3-D measurement of water flow.

The experimental setup for 3-D measurement and the spatial configuration of the three access beams on the liquid flow are shown in Figure 18. A glass pipe with a 2 mm inner diameter was set slightly tilted with respect to the optical stage (θ = 10.00° and ψ = 7.25° in Figure 18) and the velocity of water flow containing PS particles (110-nm diameter, 1 wt% concentration) was controlled precisely by the syringe pump. The water flow rate was controlled to give laminar flow whose radial velocity distribution obeyed Poiseuille’s law with maximum velocity at the center of the glass pipe. The maximum flow velocity along the central axis was estimated to be 76.32 mm/s assuming Equation (16), where a volumetric flow rate was Q/t = 479.53 mm^3^/s. In the present experiment using the same laser as Figure 8, the direction of the *f*_c,3_ beam was set along the Z-axis, while the *f*_c,1_ and *f*_c,2_ beams were in the YZ- and XZ-planes, respectively. The crossed beam-focus of the three access beams was adjusted to the central axis of the glass pipe.

**Figure 19.**
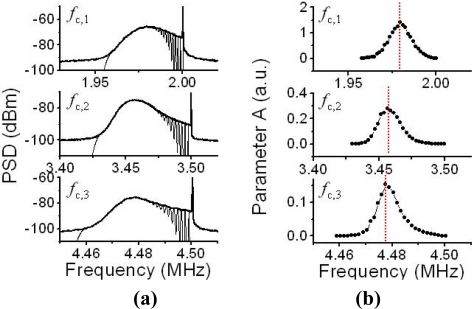
**(a)** Observed power spectra around the three carrier frequencies (bold curves). Fitting results constructed by the summation of 30 Gaussian spectra, whose intensity peaks were equally spaced in frequency, are also shown by thin curves. **(b)** Dependence of parameter A on Doppler-shift frequency used for the best fits of the experimental power spectra, as shown in (a) (reprinted with permission from [41]; © 2009, Optical Society of America).

Observed power spectra around the three carrier frequencies of the laser output, which were modulated by feedback scattered fields from moving PS particles in water flow, are shown in Figure 19. Peculiar asymmetric power spectra, reflecting a radial velocity distribution obeying Poiseuille’s law, which can be fitted by Equation (17) are seen. Indeed, the observed power spectra were found to be fitted remarkably well by the summation of Gaussian spectra given by Equation (17), as depicted by the fitting curves in Figure 19(a). The parameter *w_i_*, which is proportional to *v_i_*, was assumed to be *w_i_* = 574,100*v_i_* as given in 4.2.2.

The dependence of a fitting parameter of *A_i_* on the Doppler-shift frequency is shown in Figure 19(b). It is obvious from this Figure that *A_i_*-values peak at different frequencies for the three access beams. This may imply that the scattered light intensity was maximum at these peaks, *i.e.*, Doppler-shift frequencies, for the three access beams impinging on the liquid flow from different directions because *A*_i_ is proportional to the intensity of light scattered from particles back into the laser. These peak frequencies were expected to coincide with the Doppler-shift frequencies derived from the maximum flow velocity along the central axis of the glass pipe, assuming the impinging angles of the three access beams, since the focal position of the three access beams was adjusted to the center of the glass pipe where the effective plane-wave scattering occurred as the LDV experiment using a rotating cylinder experiment tells us [41].

Therefore, the velocity components of water flow in the center of the pipe along three orthogonal axes, X, Y, and Z, can be calculated from the three peak frequencies in Figure 19(b), and the corresponding velocity vector, *i.e.*, flow direction, can be determined by the trigonometric method. Results are summarized in Table I.

The experimental velocity vector, *i.e.*, flow direction, coincides with that expected for the experimental setup within 6% accuracy.

## 5. Microanalysis of Brownian Particles

The coherent nature of laser light has been utilized in dynamic light-scattering (DLS) methods [73] for characterizing the motion of small particles in suspension, including gasses, liquids, solids, and biological tissues [74–77]. The DLS approach to measuring diffusion broadening of scattered light from moving small particles can be used to extract useful information about particles in Brownian motion and to determine their size.

One scheme for implementing the DLS method measures the fluctuation in intensity of scattered light passing through a small pinhole, which represents the beat signals of Doppler-shifted fields scattered by different particles. The particle sizes and their distribution are estimated based on analysis of a long time-series of intensity variations given by an autocorrelation function [73–76].

Another scheme measures the beat signals between a reference light field and a scattered light field using an optical interferometer, in which a frequency shifter in one arm creates a frequency-shifted field, and an unshifted reference beam in the other arm acts as a local oscillator field. In this heterodyne detection scheme, the particle size is estimated based on the broadening of the spectrum [77].

These DLS methods developed so far have not been suitable for the quick measurement of Brownian motions of particles themselves. In this section, the application of a self-mixing thin-slice solid-state laser to quick and accurate sizing of an extremely small amount of Brownian particles in water is addressed. Also, the net motion of many Brownian particles is explored by the analysis of the demodulated signal of laser intensity fluctuations which can be done only in the self-mixing laser DLS.

### 5.1. Particle Sizing

**Figure 20.**
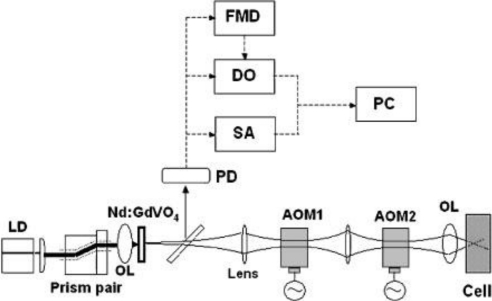
Experimental setup. PD: photoreceiver, AOM: acousto-optic modulator, OL: microscope objective lens, SA: spectrum analyzer, DO: digital oscilloscope, FMD: frequency-modulated wave demodulator, PC: personal computer (reprinted with permission from [39]; © 2006, The Japan Society of Applied Physics).

The experimental procedure is shown in Figure 20, where a 3 at.% Nd-doped 1-mm-thick Nd:GdVO_4_ laser with coated end mirrors was used as a self-mixing laser similar to 3-D measurements in Section 4.

A collimated beam from the LD operating at 808 nm was passed through a pair of anamorphic prisms and focused onto a laser crystal. A part of the laser beam (4%) was sent to an InGaAs photoreceiver (New Focus 1811: DC-125MHz) connected to a spectrum analyzer (Tektronics TDS540D: DC-3 GHz).

A 10 mm^3^ scattering cell was made of fused quartz and filled with water containing polystyrene latex (PS) spheres. The sample containing 262-nm standard spheres with 0.5% concentration in water was purchased from Estapor. The average mean diameter measured by a transmission electron microscope (TEM) in the company is 262-nm with the coefficient of variation (CV) ≤ 3%. Dilute samples with concentrations ranging from 0.05 to 50 ppm were prepared, in which distilled and tripedeonized water with an electric conductivity lower than 18.3 μS/m was used.

**Figure 21.**
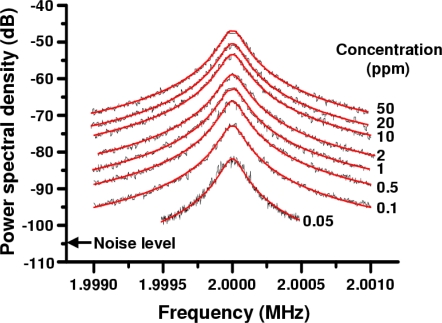
Power spectra of the modulated Nd:GdVO_4_ laser output for different concentrations of 262-nm polystyrene latex particles in water. Fitting curves are shown by red lines (reprinted with permission from [39]; © 2006, The Japan Society of Applied Physics).

Example power spectra of the modulated signal of the Nd:GdVO_4_ laser are shown in Figure 21 for different concentrations of 262 nm polystyrene latex spheres in water at temperature of 25 ± 1 °C. 262 nm particles were utilized to identify the measurable low concentration limit in the present system, since the light scattering efficiency peaks around this size at λ = 1,064 nm as Rayleigh-Debye theory predicts [38,78].

The averaged spectrum of 100 power spectra is shown for each concentration. All the power spectra were well fitted by the following Lorentz function similar to the case of heterodyne detection in the conventional dynamic light scattering method using an interferometer [37,38]:

(18) $$I(k,\omega )=A\frac{k^{2}D}{{\left(\omega -2\omega_{\mathrm{AOM}}\right)}^{2}+{\left(k^{2}D\right)}^{2}}$$

(19) $$D=\frac{k_{B}T}{3\pi \eta a}$$

where *A* is a proportionality constant related to the amplitude of the light scattered by the Brownian particles (the weak quantum noise of the laser used was neglected), *ω* is the angular lasing frequency, *D* is the diffusion constant, *a* is the diameter of the Brownian particles, *η* is the liquid medium’s coefficient of viscosity, and *k_B_T* is Boltzmann’s factor. The *k* is the magnitude of the scattered wave vector:

(20) $$k=\left(\frac{4\pi n}{\lambda }\right)\text{sin}\left(\frac{\theta }{2}\right)$$

where *n* is the refractive index of the liquid medium, λ is the wavelength of the laser, and *θ* is the scattering angle (*θ* = *π* in the self-mixing scheme) [37,38].

**Figure 22.**
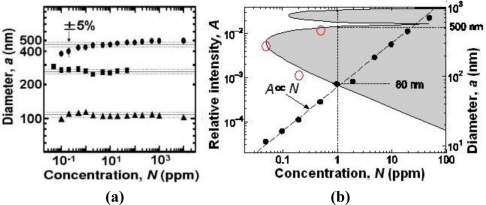
**(a)** Diameters obtained by a Lorentz curve fitting for 107-, 262- and 474-nm PS particles. **(b)** Proportionality constant (*i.e.*, relative scattered light intensity) as a function of the concentration. The gray zone indicates the concentration region where an accurate particle sizing is possible for different particle diameters (reprinted with permission from [39]; © 2006, The Japan Society of Applied Physics).

Figure 22(a) shows measured particle diameters, *a*, for 107, 262 and 474 nm PS particles as a function of concentration. The measured diameter coincides with the real value within 5%, and the linear dependence of scattered light intensity on concentration holds over a wide range of concentrations. A proportionality constant, *A*, which corresponds to scattered light intensities from particles, for the 262 nm PS particle is plotted by solid circles as a function of the concentration, *N*, in Figure 22(b). The measurable limit of concentrations for an accurate sizing was small, namely, 0.05 ppm (*i.e.*, 5 × 10^−6^ wt.%) for 262 nm particle. Measurements for 474-nm and 107-nm particles revealed the measurable limit of concentrations of 0.6 ppm and 0.2 ppm, respectively, as indicated by red sold circles in Figure 22(b).

Based on our systematic measurements of scattered light intensity dependence on particle diameters (20–500 nm), which obeys modified Rayleigh-Debye equation [38], and the measurable intensity limit of *A* = 3.5 × 10^−5^ in the present system [39], the lower concentration bound is calculated as a function of the diameter. An accurate sizing is possible in the gray region in Figure 22(b), where the lower bound is below 1 ppm for particles with diameters of 80 nm < *d* < 500 nm.

**Figure 23.**
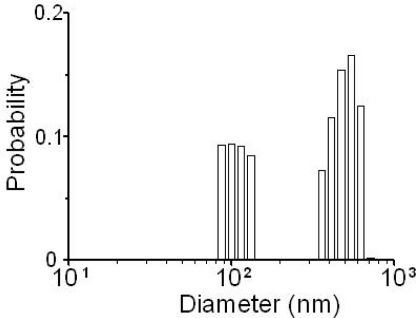
Particle size distribution obtained by SDV analysis of the measured power spectrum of a 1:1 mixed sample, consisting of 115 nm diameter PS particles in water with 0.1 wt% concentration and 474 nm diameter PS particles in water with 0.2 wt% concentration (reprinted with permission from [38]; © 2006, Optical Society of America).

The histogram analysis for mixed particles in suspension was also successfully carried out directly from measured power spectra by the truncated singular value decomposition (TSVD) method [79], not from autocorrelation functions of intensity fluctuations of a scattered light as demonstrated in usual DLS techniques. The detailed analysis procedure is given in Appendix. It was found that a reliable particle size distribution is attained. An example result for the sample containing 115 nm and 474 nm particles is shown in Figure 23.

### 5.2. Net Motion of an Ensemble of Many Brownian Particles Captured with a Self-Mixing Laser

The theories of Brownian motion have been fruitfully exploited in a multitude of phenomena not only in physics, chemistry, and biology but also physiology, economics, sociology, and politics. Among various aspects, there has been growing interest in generalizing the one-particle result to many particles in Brownian motion [80,81]. The motivation behind the study in this section arises from the general question in a wide range of disciplines: What kind of behavior arises as the overall dynamics of many individual Brownian particles within the scale of vision of the observer? This section describes an experimental approach to the presentation of the net motion of many independent Brownian particles in a dilute suspension (namely, overall dynamics) and a promising application of self-mixing laser metrology toward accurate measurement of diffusion constants of real particles from the mean-square displacement of a single ‘virtual’ particle in net motion.

#### 5.2.1. Experimental Results: Modulated and Demodulated Signals

In order to capture such overall dynamics of many Brownian particles, the dynamic light scattering method with a self-mixing thin-slice laser was employed. In this scheme, the laser itself is intensity-modulated by beat signals between the lasing field and a frequency-shifted scattered field through the interference of the two fields. The distinct advantage of our self-mixing scheme over any other method [73–75] is that we can demodulate the laser intensity fluctuations through a simple frequency-modulated (FM) wave demodulation circuit [29,37,39] and identify in real time the overall dynamics of many Brownian particles with high time resolutions.

**Figure 24.**
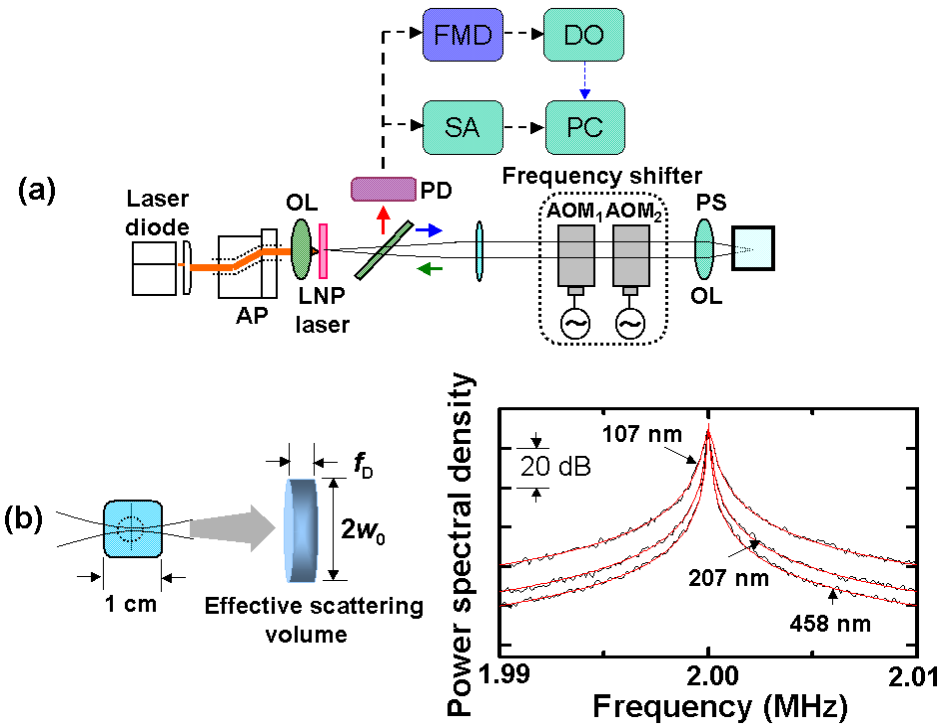
**(a)** Experimental setup of the self-mixing laser for observing net motion of Brownian particles suspended in water. AP: anamorphic prism pair, PD: photodiode, SA: spectrum analyzer (DC to 3 GHz), DO: digital oscilloscope (DC to 500 MHz), FMD: FM demodulation circuit, OL: microscope objective lens, PS: polystyrene latex cell. **(b)** Power spectra of modulated laser intensity (reprinted with permission from [42]; © 2009, American Institute of Physics).

The experimental setup is the same as Figure 20, where the laser-diode-pumped 0.3-mm-thick LiNdP_4_O_12_ (LNP) laser was employed in this experiment as shown in Figure 24(a). Example power spectra of modulated intensities obtained for 107 nm, 207 nm, and 458 nm particles (concentrations: 1 wt %) are shown in the right of Figure 24(b), where the average spectrum of 100 power spectra is shown. Within the effective scattering volume (*i.e.*, imaging field), *V*_s_ = π*w*_0_^2^*f*_D_ (*w*_0_: spot size, *f*_D_ = λ/2(*NA*)^2^ = 8.5 μm: focal depth), a sufficient number of particles existed, *i.e.*, *N* ∼2.1 × 10^3^, 2.3 × 10^4^, 1.7 × 10^5^ for 458, 207 and 107 nm particles, respectively, assuming a measured spot size of *w*_0_ = 20 μm. Here, the relatively thin effective scattering region along the laser axis with respect to the beam diameter was formed as depicted on the left of Figure 24(b). Measured power spectra are well fitted by Lorentz function given by Equation (18). Next, let us investigate the net motion of an ensemble of Brownian particles, *i.e.*, motion of an effective medium at the center of mass of particles (called a “virtual particle” hereafter) within the imaging field by demodulating the laser output intensity. The FM-wave demodulation circuit has a maximum amplifier gain of 20 dB and a 3-dB bandwidth of 111 kHz. The demodulated output voltage from the FM demodulator is proportional to the velocity of a single virtual particle along the laser axis, *v*_x_(t), in the form of *V*_o_(t) = *C* (2*v*_x_(t)/λ).

**Figure 25.**
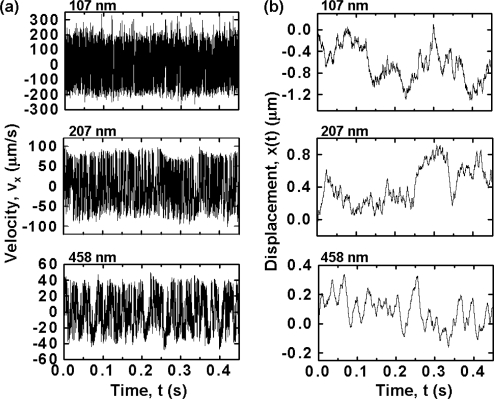
**(a)** Temporal velocity variations in velocity, *v*_x_(t), for particles with diameters of 107, 207, and 458 nm. Temporal resolution: 10 μs; **(b)** The corresponding displacement variations, *x*(t) (reprinted with permission from [42]; © 2009, American Institute of Physics).

Here, the slope, *C*, is determined by the voltage versus frequency relationship around the carrier frequency of the FM demodulator we used, which depends on the amplifier gain of the circuit. Temporal variations in calibrated velocities for different particles and the corresponding displacements, $x(t)=\int_{0}^{t}v_{x}(t)\mathrm{dt}$, are shown in Figures 25(a) and 25(b), respectively, where the observation time interval was 10 μs, *i.e.*, a sampling rate of 100 kHz which is lower than 3-dB bandwidth of 111 kHz of the FM-demodulator.

Since the virtual particle is moving at the velocity given by the vector sum instantaneous velocities of many individual particles, its velocity *v*_x_(t) is considered to be much smaller than those of individual particles. Indeed, the root mean-square velocity of a virtual particle, which is calculated from the long-term velocity variation shown in Figure 25(a), was $v_{\text{rms}}*=\sqrt{<v_{x}{\left(t\right)}^{2}>}=110$, 46, 21 μm/s for 107, 207, and 458 nm particles, respectively, *i.e.*, three-orders of magnitude smaller than those of real particles: $v_{\text{rms}}=\sqrt{k_{B}T/m}=7.97$, 2.98, and 0.91 cm/s.

The power spectra of the velocity fluctuations shown in Figure 25(a) indicate Lorentz-type of functions just like a single Brownian particle, which exhibits random motion known as the Ornstein-Uhlenbeck (OU) process resulting from the following standard Langevin equation [82]:

(21) $$m*\frac{{\mathrm{dv}}_{x}(t)}{\mathrm{dt}}=-\gamma *v_{x}(t)+F_{\mathrm{thermal}}(t)$$

where $F_{\mathrm{thermal}}(t)=\sqrt{2k_{B}T\gamma *}\xi (t)$ is a random thermal force with < *ξ* (*t*) >=0 and < *ξ*(*t*)*ξ*(*t*′) >= *δ*(*t* – *t*′). Here, m* and γ* are the mass of a virtual particle and friction coefficient of a virtual liquid, respectively.

**Figure 26.**
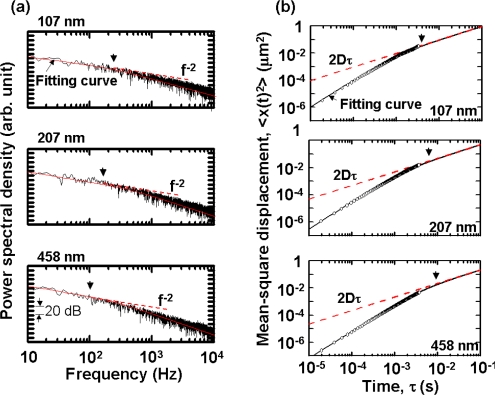
**(a)** Power spectra of the motion of virtual particles; **(b)** Mean-square displacements, which can be fitted by the sold lines. The ensemble average of 200 data sets extracted from the time series of Figure 25(b) was calculated for each value of τ. Theoretical mean-square displacements of real particle in pure Brownian motions are indicated by dashed lines (reprinted with permission from [42]; © 2009, American Institute of Physics).

Figure 26(a) shows power spectra of the temporal evolutions of the displacement, *x*(t), which is given by *S*(*ω*) ∝ 2D* / *ω*^2^ (1+*ω*^2^*τ_m_* *^2^) from Equation (21), where τ_m_* = m*/γ* = ρ* a*^2^/18η* (ρ*: density) is called the *persistence* time, and characterizes the time for which a given velocity is “remembered” by the system and D* is the diffusion constant of the virtual particle. Note that the roll-off frequencies from the *f*^−2^-type random walk toward asymptotic *f*^−4^ behavior, which are indicated by arrows, appear at frequencies that are 5–6 orders of magnitude lower than those for real particles. Here, fitting curves are indicated by solid lines. Because these roll-off frequencies are much lower than the 3-dB bandwidth of 111 kHz, the observed behavior is considered to be generic in the net motion, being free from measurement limitations of the instrument. The roll-off frequency was measured to decrease with decreasing the particle concentrations and the net motion approached pure random walk.

Figure 26(b) shows the mean-square displacement, <*x*(τ)^2^> = <[*x*(t + τ) − x(t)]^2^>, where τ is a variable time delay. The experimental values (open circles) were fitted surprisingly well by the following equation derived from Equation (21) [81,82]:

(22) $$<x{\left(\tau \right)}^{2}>=2D*[\tau -\tau_{m}*\{1-\text{exp}(-\tau /\tau_{m}*)\}],$$

as indicated in Figure 26(b), where fitting curves for virtual particles and theoretical results for real particles are also shown by solid and dashed lines, respectively.

Relevant diffusion coefficients D*, which respectively coincide with the theoretical values for real particles *D*, were found to be attained for virtual particles. This is reasonable because the power spectra of modulated signals of 12.8-ms-long frames for accurate sizing of real particles shown in Figure 24(b) should hold for virtual particles for observation time scale shorter than the frame length in Figure 26(b). On the other hand, the persistence times of virtual particles, τ_m_* = 0.43, 0.78, and 1.67 ms, obtained from curve fittings are 5–6 orders of magnitude longer than those of real particles with 107, 207 and 458 nm diameters: τ_m_ = 0.71, 2.67, and 13.1 ns, respectively. These τ_m_*-values also coincided well with those obtained from the curve fitting shown in Figure 26(a), and the roll-off toward the pure random walk appeared at τ_r_ = 1/*f*_r_, which are indicated by arrows in Figure 26(b). It is obvious that the high-velocity components were substantially suppressed, as shown in Figure 26(a), and a drastic reduction in the mean-square displacement compared with purely diffusive motion arose on the observation time scale shorter than τ_r_ in Figure 26(b).

The mean-square displacement was calculated by using long-term time series (10 seconds) of demodulated signals measured at a time resolution of 200 μs, in which the gradient was decreased gradually as τ increases similar to Figure 26(b) and the pure Brownian random walk <*x*(τ)^2^> = 2*D*τ was confirmed to appear in the regime of τ > 10 ms.

Such extremely long persistence times cannot be explained in terms of collective diffusion due to hydrodynamic interactions among particles [81]. Also, the non-diffusive motion of a Brownian particle featuring elongate persistence times due to the liquid's inertia, which is expressed by Equation (3) of Reference [83], indicated deviations from the <*x*(τ)^2^> = 2*D*τ relation around τ = 0.1, 0.2 and 0.5 μs for 107, 207 and 458 nm particles, respectively. Consequently, the observed behavior is considered to be inherent in the overall dynamic of Brownian particles.

The similar overall dynamics, which obeys Equation (21), have always appeared for Brownian particles with different diameters (*i.e.*, 115, 262 and 474 nm). The theoretical treatment is anticipated for explaining the OU process with elongate persistence times and the link between single-particle and overall dynamic, including the effects of number of particles moving within the imaging field and observation time intervals on the net motion.

#### 5.2.2. Scaling of Physical Parameters between Real and Virtual Particle Suspensions

**Figure 27.**
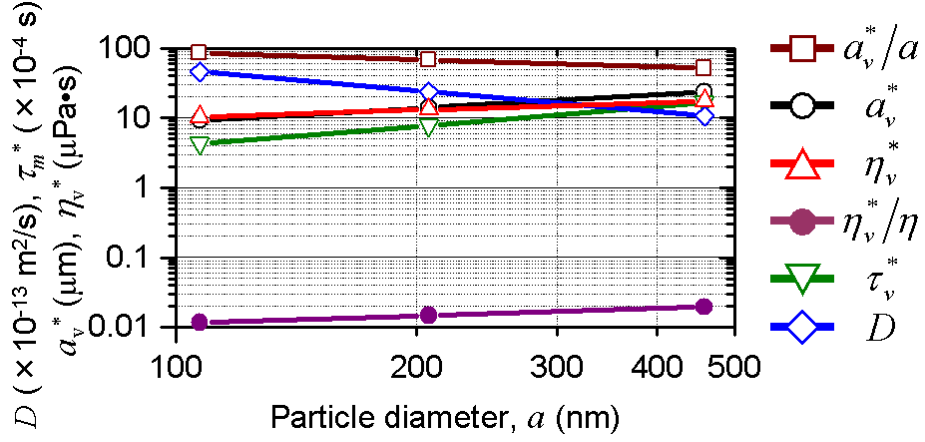
Physical parameters of virtual turbid media as a function of the particle diameter, which is determined from the measured persistence times (reprinted with permission from [42]; © 2009, American Institute of Physics).

Let us finally examine physical parameters of virtual particles. Provided that the friction constant should be the same for real and virtual particles to insure the same diffusion coefficient for both particles, virtual particle's diameter, a*, and virtual liquid's viscosity, η*, can be given by the following relations: a* = (*ρτ_m_* * / ρ*τ_m_)^1/3^ *a* = (*τ_m_* * / *ρ_s_* *τ_m_)^1/3^ *a*, η* = (*ρτ_m_* * / ρ*τ_m_)^–1/3^ *η* = (*τ_m_* * / *ρ_s_* *τ_m_)^–1/3^ *η*, where ρ* and *ρ*_s_* are density and specific gravity with respect to water of a virtual particle. The experimentally determined persistence times of virtual particles and the resultant normalized diameters, *a*_v_* ≡ a*ρ_s_* ^1/3^, and virtual suspension's viscosities, *η*_v_* ≡ η*ρ_s_*^−1/3^ for 107-, 207-, 458-nm particles are shown in Figure 27. Physical parameters of virtual turbid media are found resemble those expected in low-density, large-diameter particles (e.g., aerosol of several tens of microns) moving in gas.

From the application viewpoint, the present self-mixing laser dynamic light scattering method provides a promising for measuring diffusion coefficients with high accuracy from demodulated signals measured at appropriate time intervals without using a high resolution wide-band spectrum analyzer. Promising applications could include *in-situ* measurement of cohesion and segregation process of colloidal particles over times.

#### 5.2.3. 3-D Trajectory of the Brownian Motion of a Virtual Particle

**Figure 28.**
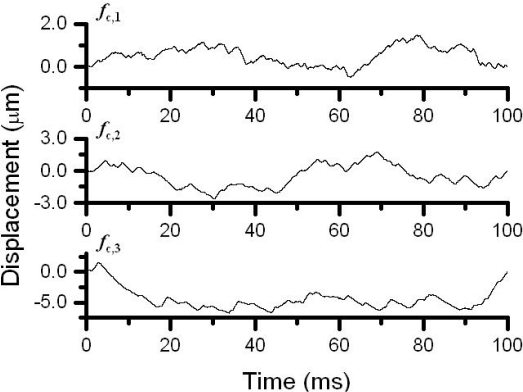
Temporal evolutions of displacements of a virtual particle along the directions of the three access beams (reprinted with permission from [41]; © 2009, Optical Society of America).

The 3-D motion of a virtual particle was captured by using the 3-channel self-mixing Nd:GdVO_4_ laser flowmetry scheme mentioned in 4.2.5, where three access beams were impinged into the scattering cell containing PS particles in water. The output voltage from each channel of the 3-channel FM-demodulator is proportional to the instantaneous velocity of a single virtual particle within the limited field of vision along the axis of each access beam. The displacement of a virtual particle along each direction, which is calculated by integrating the velocity over time, *i.e.*, *D*(*t*) = ∫ (*v*(*t*) –*v̄*)*dt*, is shown in Figure 28.

**Figure 29.**
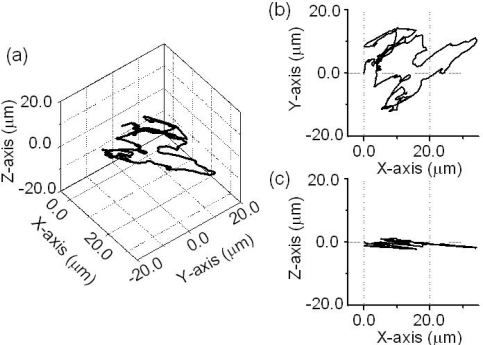
Reconstructed trajectory of the motion of a virtual particle: **(a)** bird’s eye view; **(b)** projected onto the XY plane, and **(c)** projected onto the XZ plane (reprinted with permission from [41]; © 2009, Optical Society of America).

Using the trigonometric method, we reconstructed the three-dimensional movement of the virtual particle in the crossed beam-focus. The resultant trajectory of the virtual particle is shown in Figure 29. The reconstructed trajectory indicates a random walk in three dimensions, which is characteristic of the nature of Brownian motion. It should be noted that the movement along the Z-axis, which is the axis of access beam *f*_c,3_, was smaller than the movement within the XY-plane. This peculiar nature of the motion of a virtual Brownian particle captured by the present 3-D self-mixing laser dynamic light scattering scheme is interpreted as follows: When the region of crossed beam-focus was adjusted within the scattering cell such that effective plane-wave scattering occurred for accurate sizing, as shown in Figure 24, the effective scattering volume is approximately given by *V*_s_ = π*w*^2^*f*_d_, where *w* is the focused beam spot size and *f*_d_ = λ/2(*N.A.*)^2^ is the focal depth of the objective lens. In the present case, the estimated focal depth, *f*_d_ = 8.5 μm, was much shorter than the focused beam diameter of 36 μm. Consequently, the movement of the virtual particle along the Z-axis is considered to be limited by the narrow field of vision of particles seen by laser beams as shown in Figure 29(c).

If we apply the present set-up to anisotropic media, e.g., liquid crystals, we expect to capture quantitatively different dynamic behaviors of molecules along three orthogonal directions.

## 6. Application of Self-Mixing Solid-State Laser to Biological Species Measurement

Optical oceanography has progressed to the point where it is necessary to know the inherent optical properties of individual marine particles. Suspended particles determine the apparent and inherent optical properties of seawater, such as ocean color. Recently, several optical techniques have been developed to measure the spatial and temporal variability of the inherent and apparent optical properties of particles suspended in seawater [83–90]. For example, a flow cytometer can measure size distributions and complex refractive indices of phytoplankton in Brownian motion from the transmitted and scattered light [91–94]. Laser Doppler vibrometry (LDV) spectroscopy can also measure swimming velocities of mobile phytoplankton [86]. In this section the application of self-mixing laser Doppler metrology to real-time measurements of the motion of phytoplankton toward dynamic characterization of biological species, including accurate sizing and velocity distributions are described [38,43].

### 6.1. Quick and Accurate Sizing of Living Phytoplankton

The self-mixing DLS method employing the 0.3-mm-thick LNP laser mentioned in Section 5.2 was used to perform quick particle sizing and characterize the motion of two types of phytoplankton, *Nannochloropsis oculata* and *Tetraselmis tetrathele. Nannochloropsis* is spherical, with a diameter of about 2.7 μm (measured using a Coulter counter); it cannot move voluntarily in seawater. *Tetraselmis* is nearly spherical and has a diameter of about 7.0 μm; it can move quickly using a flagellum, *i.e.*, voluntary motion, in seawater. The scattering cell described in Section 5.1 was filled with seawater containing one of these two types of phytoplankton and the same focusing objective lens was used.

**Figure 30.**
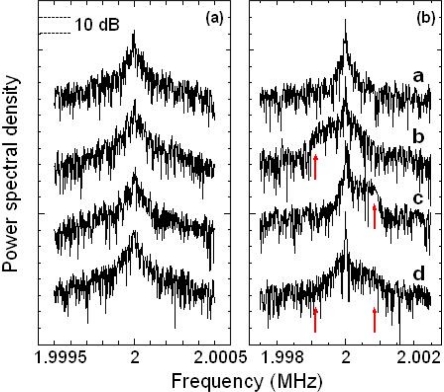
Power spectra of modulated outputs observed at different times for **(a)** *Nannochloropsis oculata* and **(b)** *Tetraselmis tetrathele*. The Supplementary Material demonstrates time-dependent power spectra and sounds from *Nannochloropis* (1.5 Mbytes) and *Tetraselmis* (1.55 Mbytes) in seawater. Note that the sound was obtained by delivering the demodulated output signal (velocity) to the speaker, which possesses an integral function and produces the sound corresponding to the movement of plankton (reprinted with permission from [43]; © 2009, Optical Society of America).

Figures 30(a) and 30(b) show time-dependent power spectra for *Nannochloropsis* and *Tetraselmis*, respectively. The power spectrum observed for non self-mobile *Nannochloropsis* maintains a Lorentz shape in time, implying Brownian motion. On the contrary, the power spectrum for self-mobile *Tetraselmis* changes in time: Broad frequency components resulting from self-motion of part of plankton are superimposed occasionally on a Lorentz spectrum, **a** in Figure 30(b), which reflects Brownian motions, resulting in shoulders on both sides of the carrier frequency as indicated by arrows in **b–d** in Figure 30(b).

**Figure 31.**
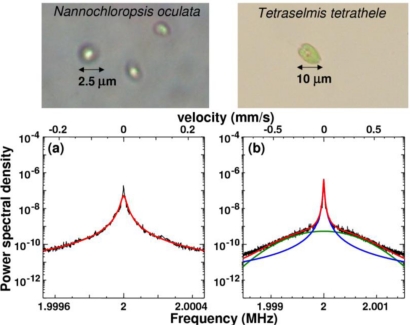
Optical microscope images and averaged power spectra of modulated output signals. **(a)** *Nannochloropsis oculata*; black line: experiment, red line: curve fitting; **(b)** *Tetraselmis tetrathele*; black line: experiment, red line: curve fitting by the summation of Lorenz (blue line) and Gauss (green line) functions.

To provide more insight into the observed power spectra, let us characterize the measured average power spectra in a statistical way. From curve-fitting of the averaged power spectrum for non self-mobile *Nannochloropsis* shown in Figure 31(a), the average diameter was estimated to be 2.2 μm, which is close to the diameter measured by a Coulter counter. The averaged power spectrum for self-mobile *Tetraselmis* is shown in Figure 31(b), where the spectrum is decomposed into two components by a curve-fitting, assuming the superposition of a Lorentz (*i.e.*, Brownian motion) and a Gaussian (self-motion) spectrum. The measured power spectrum is well-reproduced by such a curve-fitting as shown by the red curve, in which corresponding Lorentz and Gaussian components are also depicted by blue and green curves, respectively. From the using the relation *f*_D_ = 2 *v*_z_/λ, we can estimate a distribution of the velocity along the laser axis *v*_z_ of Brownian or self-motion. An estimated speed is given in the upper horizontal axis.

Detailed characterization of peculiar dynamic behavior in the voluntarily motion of *Tetraselmis* plankton, represented by occasional appearance of ‘shoulder’ structure indicated by red arrows, and precise quantitative velocity analysis of motions of self-mobile phytoplankton will be discussed in the following sections.

### 6.2. Analysis of the Shoulder-Shaped Power Spectrum

**Figure 32.**
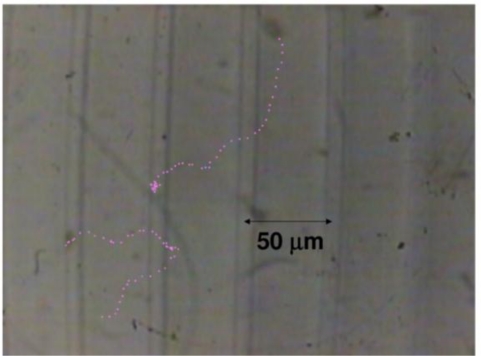
Trajectory of the motion of *Tetraselmis tetrathele* captured by an optical microscope with a CCD camera. The time interval of pink dots is 10 milliseconds. See the Supplementary Material (2.463 Mbytes).

Before the self-mixing LDV experiment, in order to estimate the velocity of phytoplankton, the motion of the *Tetraselmis* was observed directly with an optical microscope having a digital system (charge coupled device, CCD), and the temporal image was sent to a computer for analysis. A typical motion, featuring occasional circulations, is shown in Figure 32. The time interval of recorded images was 10 ms. The imaging field of the microscope was about 100 μm × 100 μm, and the number of living phytoplankton in this area was restricted to one or two in the observation time scale. The average speed of translational motion for the *Tetraselmis* was estimated to be *v*_avg_ = 0.36 mm/s from the recorded image data. See the corresponding Supplementary Material.

The relationship between power spectrum shape and particle velocity was investigated to understand the physical meaning of the shoulder-shaped power spectrum. As indicated in section 4.2, the power spectrum of light scattered from small particles moving uniformly is a Gaussian spectrum whose intensity and width depend on the transient (dwelling) time of particles across the incident light beam:

(23) $$I(\omega )=A_{G}\;\text{exp}\left\{-\frac{{\left(\omega -2\pi f_{D}\right)}^{2}}{w^{2}}\right\}$$

where *ω* is the angular frequency. Empirical parameters *A_G_* and *w* are the amplitude and width of the Gaussian spectrum relating to the velocity of the particle, respectively. *f_D_* is the Doppler shift frequency of scattered light given by Equation (14).

Note that the shoulder-shaped power spectrum is occasionally superimposed on the Lorentz spectrum, which reflects the Brownian motion, in time for the self-mobile phytoplankton in seawater, indicated by red arrows in Figure 30(b), is similar to that for the dilute sample flow through a small-diameter glass pipe shown in Figure 12. Therefore, the shoulder-shaped power spectrum for the self-mobile phytoplankton in seawater is expected to reflect the velocity distribution of the self-mobile phytoplankton, and the velocities of self-moving phytoplankton can be determined quantitatively by analyzing the power spectrum using the sum of Gaussian spectra with continuously changing widths and peak frequencies expressed by Equation (17) similar to those for dilute sample flow.

**Figure 33.**
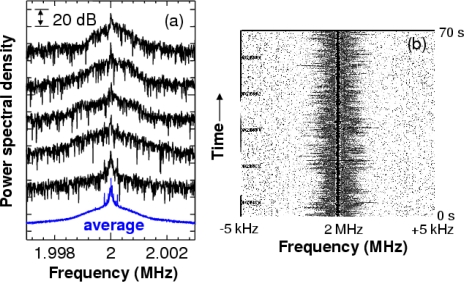
**(a)** Power spectra and **(b)** time dependence of power spectra (Joint Time-Frequency Analysis; JTFA) for *Tetraselmis tetrathele* in seawater at different times (reprinted with permission from [43]; © 2009, Optical Society of America).

Figure 33(a) shows time-dependent power spectra and the long-term average power spectrum. Figure 33(b) shows a joint time-frequency analysis (JTFA) of the power spectra. The shoulder-shaped power spectrum, which reflects the self-mobility of phytoplankton, is occasionally superimposed on the higher or lower frequency side of the carrier frequency 2*f*_AOM_ of a Lorentz spectrum, which reflects the Brownian motion.

The frequency span of the spectrum analyzer was set to 5 kHz in these measurements, so the time interval of the update of the power spectrum, *i.e.*, time resolution, was 128 ms. On the other hand, the estimated focal depth and focused beam diameter were 8.5 μm and 36 μm, respectively [41]. From the observation with a microscope having an imaging field of 100 μm × 100 μm, it was noticed that one phytoplankton occasionally moved across this focus area of the self-mixing LDV. In this case, it is quite possible that the velocity of self-mobile phytoplankton changed in time and this velocity change of one phytoplankton, moving across the focus area within the time resolution, resulted in the shoulder-shaped power spectrum in the observation time scale.

To obtain the phytoplankton velocities, least squares curve fitting was performed for the power spectrum using the sum of single Lorentz and multiple Gaussian spectra:

(24) $$I(\omega )=A_{L}\frac{k^{2}D}{{\left[\omega -2\pi (2f_{\mathrm{AOM}})\right]}^{2}+{\left(k^{2}D\right)}^{2}}+\sum_{i}c_{i}A_{\mathrm{Gi}}\;\text{exp}\left\{-\frac{{\left[\omega -2\pi (2f_{\mathrm{AOM}}+f_{\mathrm{Di}})\right]}^{2}}{w_{i}^{2}}\right\}.$$

where the first term indicates the Lorentz function reflecting the Brownian motion of phytoplankton: *A*_L_ is a proportionality constant related to the amplitude of the light scattered from the phytoplankton in Brownian motion, *k* is the wave vector, and *D* is the diffusion constant. The second term indicates the sum of the Gaussian spectra reflecting the velocity change of phytoplankton during the time interval of the update of the power spectrum, *i.e.*, time resolution: *c*_i_ are the weighting constants for the Gaussian spectra, and they are related to a dwelling time of the phytoplankton in the focus area in the time resolution.

**Figure 34.**
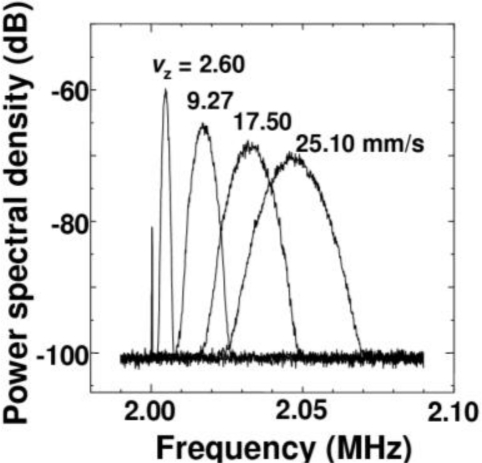
Power spectra for rotating disk with various disk velocities in the focus area (reprinted with permission from [43]; © 2009, Optical Society of America).

To perform accurate curve fitting for the shoulder-shaped power spectrum, the relationships between the velocity of the moving target and empirical parameters *A* and *w* in the Gaussian spectrum terms in Equation (24) was determined by using a rotating disk with uniform angular velocity instead of the scattering cell under the same beam focusing condition. Observed power spectra for different rotating velocities are shown in Figure 34.

**Figure 35.**
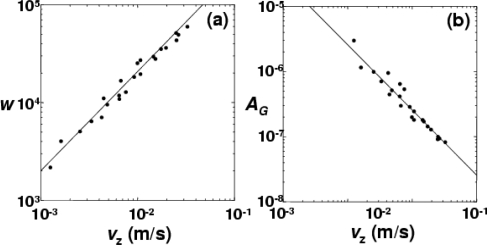
Velocity of rotating disk versus **(a)** parameter *w* and **(b)** *A_G_* (reprinted with permission from [43]; © 2009, Optical Society of America).

It is obvious that the spectrum peak decreases and spectrum width increases with increasing velocity of the rotating disk. Here, the least squares curve fitting for the power spectrum using Equation (23) was carried out. Plots of *v*_z_ versus *A*_G_ and *w* are shown in Figure 35, yielding the empirical relations of *A_G_* = 2.55 × 10^−9^ *v_z_*^−1^, *w* = 1.46 × 10^6^ *v_z_*.

**Figure 36.**
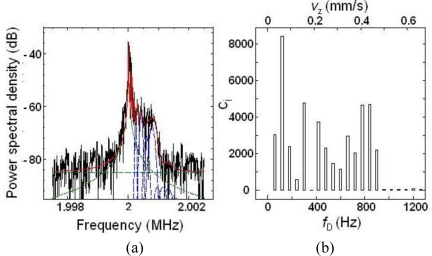
**(a)** Power spectrum for *Tetraselmis tetrathele* in seawater. The black line indicates the observed power spectrum. Green dashed and dotted lines indicate the noise level and the Lorentz term, respectively. Blue dashed lines indicate the Gaussian terms for the velocity elements. The red solid line indicates the sum of Lorentz and Gaussian terms and noise level. **(b)** *f*_D_ versus proportionality constant of each velocity element *c*_i_. (reprinted with permission from [43]; © 2009, Optical Society of America).

Figure 36(a) shows the results of the curve fitting of the power spectrum for the self-mobile phytoplankton. Figure 36(b) shows the histogram of weighting constant *c*_i_ for the velocity elements obtained from the curve fitting. From this histogram, the average velocity in the frame length of 128 ms is estimated to be [*v*_z_]_avg_ = 0.27 mm/s.

The translational motion of self-mobile phytoplankton is generally isotropic, and its velocity vector changes in time. Using LDV, the velocity vector can be obtained as Equation (14) when the angle between the directions of the probe beam and moving target is known. Thus, single-channel LDV should let us detect the self-mobile phytoplankton moving across the probe beam and measure the velocity along the probe beam axis. If we use three access beams impinge on a self-mobile phytoplankton from different directions, as demonstrated in 4.2.5 and 5.2.3, the velocity of the target along each beam axis can be measured independently. Curve fitting of measured spectra using Equation (24) and trigonometric vector calculation give the velocity vector of one phytoplankton in the crossed beam focus.

### 6.3. Physical Meaning of Long-Term Average of Power Spectra

**Figure 37.**
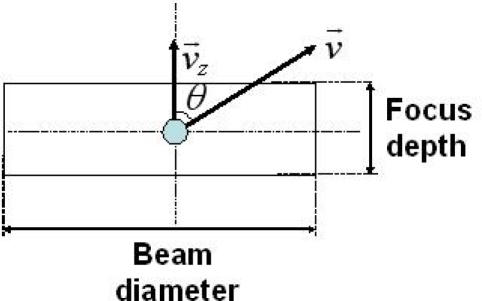
Particle movement in the focal area.

On the other hand, the average motion of self-mobile phytoplankton in an isotropic medium is expected to be obtained from the results of the *long-term* average of power spectra, considering the effect of the asymmetric “thin” focus area of the present self-mixing LDV [41]. Here, let us discuss the average power spectrum for self-mobile phytoplankton. To obtain the average motion of the self-mobile phytoplankton from the long-term power spectrum, two dimensional Monte Carlo simulation was carried out using the following procedure: one particle is placed at the center of the observation area as an ideal phytoplankton, as depicted in Figure 37, where the observation area size is assumed to coincide with the focus area of LDV (focal depth 8.5 μm, focused beam width 36 μm).

When the particle moves in the focus area, the frequency of scattered light is shifted depending on *v*_z_, so the intensity of the power spectrum is proportional to the transient (dwelling) time of the particle in the focus area. Therefore, if θ ≅ 0, the Doppler shift frequency is large, but the intensity of the power spectrum is small because the particle quickly leaves the focus area. In contrast, if θ ≅ π/2, the Doppler shift frequency is small, but the intensity of power spectrum is large. The probability of the particle’s speed having a Gaussian distribution is assumed to be:

(25) $$p(v)=\text{exp}\left\{-\frac{{\left(v_{\mathrm{avg}}-v\right)}^{2}}{2\;w^{2}}\right\},$$

where *v*_avg_ is the average speed and *w* is the speed distribution.

**Figure 38.**
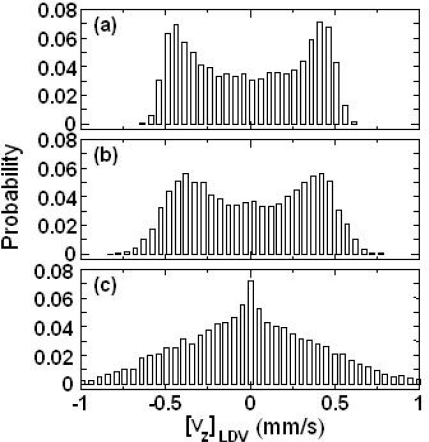
Histogram of *v_z_*_·_ *v_z_*=0.50 mm/s, **(a)** *w* = 5.0 × 10^−2^ mm/s, **(b)** *w* = 0.10 mm/s, and **(c)** *w* = 0.30 mm/s (reprinted with permission from [43]; © 2009, Optical Society of America).

The probability distribution of moving directions can be assumed to be isotropic. Then, the particle speed and direction are given by random numbers. We performed 10,000 Monte Carlo simulations of one particle with a random velocity. The histogram of *v*_z_ is shown in Figure 38. When the velocity distribution was narrow, two peaks were observed; when it was broad, only one peak was observed at *v*_z_ = 0.

**Figure 39.**
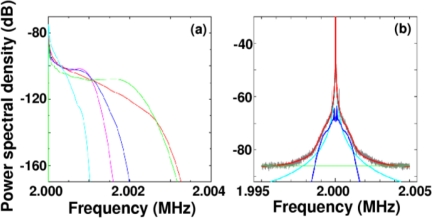
**(a)** Power spectra calculated from the sum of the Gaussian spectra for various velocity distributions: purple line (*v*_avg_ = 0.50 mm/s, *w*_s_ = 0.050 mm/s), blue line (0.50 mm/s, 0.10 mm/s), red line (0.50 mm/s, 0.30 mm/s), light-blue line (0.10 mm/s, 0.10 mm/s), and green line (1.0 mm/s, 0.10 mm/s); **(b)** Average power spectrum for Tetraselmis tetrathele in seawater.The grey line indicates the observed power spectrum. The green and light-blue lines indicate the noise level and Lorentz term, respectively. The blue line indicates the sum of Gaussian spectra (*v*_avg_ = 0.40 mm/s, *w*_s_ = 0.013 mm/s). The red line indicates the sum of Lorentz and Gaussian terms and noise level.

The sum of multiple Gaussian spectra reflecting the translational motions of phytoplankton is shown in Figure 39(a). The shape of the power spectrum depends strongly on the velocity distribution of the particles. The sum of these multiple Gaussian spectra and the single Lorentz spectrum shows a good agreement with the observed long-term average power spectrum as shown in Figure 39(b). The *v*_avg_ value of 0.40 mm/s determined by the curve fitting is close to 0.36 mm/s determined from the microscope imaging field. These results indicate that the average velocity and the velocity distribution can be calculated from the average power spectrum.

### 6.3. Real-Time Measurement of Motions of Self-Mobile Phytoplankton Toward Light

Finally in this section, the experiment of capturing motions of phytoplankton toward light with the self-mixing laser LDV scheme is summarized briefly. While, it is a hard task to measure such motions quantitatively in real-time with state-of-the-art metrology system.

**Figure 40.**
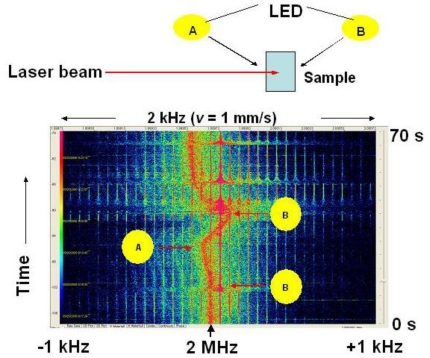
*Tetraselmis* move toward light.

The experimental setup of the cell filled with seawater containing *Tetraselmis* is depicted in the upper part of Figure 40. Two light-emitting-diodes (LEDs) A and B were set on both sides of the cell. An example JTFA signal pattern is shown in the lower part of Figure 40. When LED A (B) is switched on as indicated by the arrows, the peak frequency of LDV signal shift to the higher (lower) frequency side. Thus, it is obvious that *Tetraselmis* moves toward the light. Here, their velocity corresponding to the JTFA pattern is indicated on the upper horizontal axis of Figure 40.

## 7. Yb-YAG for Self-Mixing Laser Metrology with Enhanced Optical Sensitivity

I have reviewed several self-mixing metrology systems using such thin-slice Nd-doped solid-state lasers as LiNdP_4_O_12_ (LNP), Nd:GdVO_4_. As discussed in Section 3.1, the optical sensitivity can be enhanced in proportion to the fluorescence-to-photon lifetime ratio of the laser media. This implies that laser materials with longer fluorescence lifetimes are suitable for further enhancement of optical sensitivity.

In this section, self-mixing laser Doppler measurements using a laser-diode-pumped thin-slice Yb:YAG solid-state laser are described and it is shown experimentally and theoretically that the optical sensitivity is enhanced by two orders of magnitude as compared with that of Nd-doped solid-state lasers, under the same cavity configuration, resulting from longer fluorescence lifetime inherent in Yb atoms [96]. The successful detection of nanometer-scale vibrations of a target embedded in large-amplitude environmental vibrations, which is desired in the actual measurement circumstances, will be also shown [95].

### 7.1. Self-Mixing LDV Experiment: Comparison between Nd- and Yb-Doped Lasers

#### 7.1.1. Experimental Results

**Figure 41.**
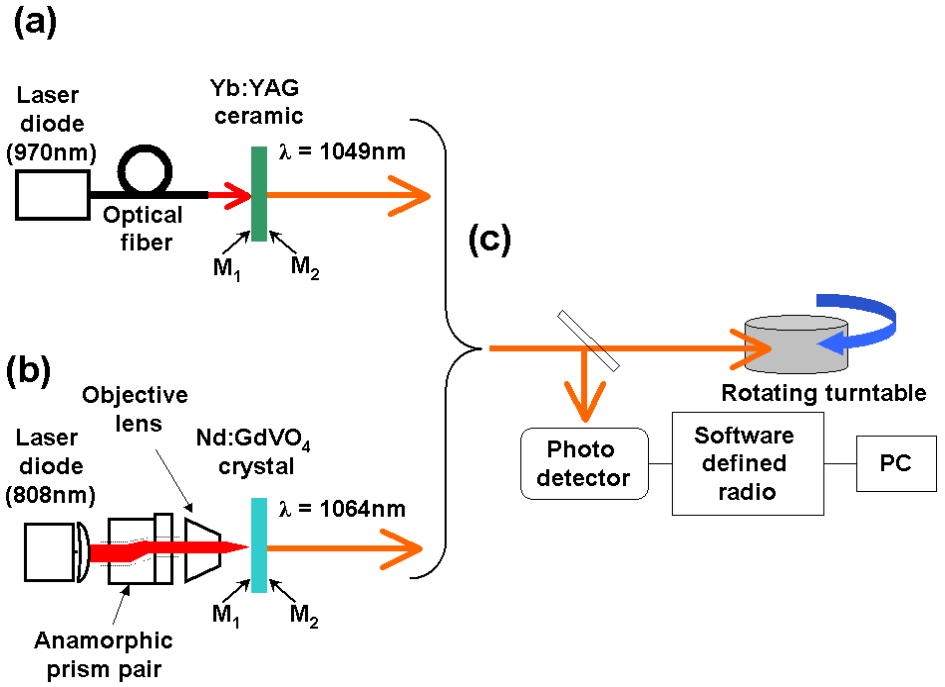
Experimental setup of a self-mixing laser Doppler velocimetry. **(a)** Yb:YAG laser pumped with an LD with a fiber pigtail and **(b)** Nd:GdVO_4_ laser pumped by an LD beam with a microscope objective lens; **(c)** Self-mixing laser velocimetry scheme using a rotating turntable made of Al.

The first experimental setup is shown in Figure 41, where the simplest self-mixing LDV experiment constructed by (a), (c) and (b), (c) was carried out by using different solid-state laser media. A 5 at.% Yb-doped 2-mm-thick Yb:YAG ceramic sample consisting of randomly distributed single-crystalline grains, whose average size was about 3.20 mm, was used and the end surfaces of this sample were coated with mirrors, M_1_ (99.8% reflectance at 1,049 nm and 95% transmittance at 970 nm) and M_2_ (98% reflectance at 1,049 nm). Pump light from an LD with a fiber pigtail, whose core diameter was 100 μm, operating at a wavelength of 970 nm, was impinged directly on M_1_, as shown in Figure 41(a). The lasing wavelength was λ = 1,049 nm and linearly polarized TEM_00_-mode operations were obtained. The threshold pump power was *P_th_* = 650mW and the slope efficiency was 50%.

For comparison, a 3 at.% Nd-doped 1-mm-thick Nd:GdVO_4_ single crystal sample with mirrors, M_1_ (99.8% reflectance at 1,064 nm and 95% transmittance at 808 nm) and M_2_ (98% reflectance at 1,064 nm), was also used. A collimated elliptical beam from the LD beam operating at 808 nm was transformed into a circular beam using an anamorphic prism pair. The beam was then focused onto M_1_ by a microscope objective lens of numerical aperture (NA) = 0.25, as shown in Figure 41(b). The lasing wavelength was 1,064 nm and linearly polarized TEM_00_-mode operations were obtained. The threshold pump power was 32 mW and the slope efficiency was 24%.

From the pump-dependent relaxation oscillation frequency, as mentioned in 4.1.2, the photon lifetime of the Yb:YAG laser was τ_p_ = 176 ps, assuming a fluorescence lifetime of τ = 900 μs, yielding the lifetime ratio of *K* = 5.11 × 10^6^. While, τ_p_ = 235 ps and *K* = 3:83 × 10^5^ as for the Nd:GdVO_4_ laser, assuming τ = 90 μs. Therefore, the *K*-value of the Yb:YAG ceramic laser is about 13.3 times larger than that of the Nd:GdVO_4_ laser, mainly owing to its longer fluorescence lifetime, and an increased optical sensitivity of the Yb:YAG ceramic laser is expected.

To confirm such an expectation and evaluate the increased optical sensitivity quantitatively, a simple laser-Doppler velocimetry experiment without AOM frequency shifters was carried out as shown in Figure 41(c), where the laser output beam was impinged on a rotating cylinder through a lens. Here, 4% of a laser beam split by a glass plate was used for monitoring and the remainder (96%) was sent to a target. In this scheme, the laser is intensity-modulated at *f*_D_ = 2*v*_z_/λ (*v*_z_ moving speed along the laser axis of a target). Both lasers were operated under the same optical feedback conditions using a common target through the same optical path. The laser output was detected by an InGaAs photoreceiver (New Focus 1811: DC-125 MHz) and analyzed by a spectrum analyzer (Advantest R3131A: 9 kHz-3 GHz) or a software-defined radio (RF-Space SDR-14: DC-30 MHz).

**Figure 42.**
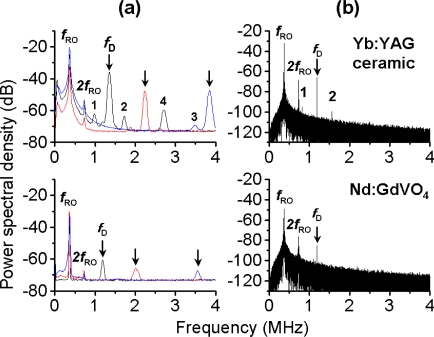
**(a)** Experimental power spectra of modulated Yb:YAG laser (upper figure) and Nd:GdVO_4_ laser (bottom figure) measured for different rotation velocities of the turntable; **(b)** Numerical results of power spectra calculated for two lasers (reprinted with permission from [95]; © 2009, The Japan Society of Applied Physics).

Typical power spectra of modulated lasers are shown in Figure 42(a) (frequency resolution: 8.138 kHz) for different rotation velocities of the turntable, in which pump powers (*i.e.*, *w* = *P*/*P_th_* values) for both lasers were adjusted such that both show the same relaxation oscillation frequency to ensure a common frequency response. It is clear that the power spectral density of the Yb:YAG laser is about 177 times larger (*i.e.*, 22.4 dB) than that of the Nd:GdVO_4_, as was expected from the 13.3-times-larger *K* value (*i.e.*, amplitude modulation index) under the same optical feedback ratio, since the power spectral density is proportional to the square of the Fourier component of the amplitude-modulated laser signal. Note that peaks 1, 2, and 3 in power spectra for the Yb:YAG laser denote relaxation oscillation sidebands, *f*_D_ ± *f*_RO_, and peak 4 indicates the second-harmonic component, 2*f*_D_.

#### 7.1.2. Numerical Result

Numerical simulations were carried out using the model equation for lasers subjected to frequency-shifted optical feedback including spontaneous emission noise, Equations (9–12), to reproduce experimental results and estimate the optical feedback ratio. Numerical results are shown in Figure 42(b), assuming *w* = 1.840 for Yb:YAG, *w* = 1.112 for Nd:GdVO_4_ and relevant *K*-values for both lasers as determined experimentally in 7.1.1. The common parameters are *m* = 10^−6^ and *f*_m_ = *f*_D_ = 1.188 MHz, where *R_f_* is the amplitude feedback ratio. Here, Gaussian white noise with zero mean was introduced as spontaneous emission to reproduce experimental noise spectra exhibiting *f*_RO_ and weak 2*f*_RO_ components. Gaussian-type frequency broadening of scattered light fields from a rotating rough surface, which results in the broadened *f*_D_ components in the experiments [Figure 42(a)], was neglected in simulations for brevity. The numerical results agree well with the experimental results, *i.e.*, an increase in power spectral density of 24 dB at *f*_D_ for Yb:YAG, and an intensity feedback ratio, 10 log*R_f_*^2^, was estimated to be −120 dB. The lowest limit of intensity optical feedback ratio for the successful measurement, which is restricted by the spontaneous emission noise, is estimated to be −154 dB from the noise level in the present experiment.

**Figure 43.**
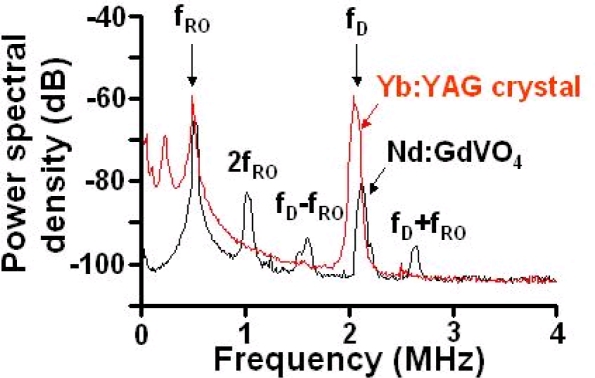
Power spectra of modulated single-crystalline Yb:YAG laser and Nd:GdVO_4_ laser in the LDV experiment.

A 2-mm-thick, 20 at.% Yb-doped single crystalline thin-slice Yb:YAG laser was also used instead of Yb:YAG ceramic and the similar enhancement of optical sensitivity was obtained. The threshold pump power was 500 mW and the slope efficiency was 43 %. A power spectrum in LDV experiment with the single-crystalline Yb:YAG laser is shown in Figure 43, in comparison with that of Nd:GdVO_4_ laser. The enhancement of 23 dB was attained. The experimentally determined key parameters are summarized in Table 2.

### 7.2. Real-Time Nano-Scale Vibration Measurement in the Presence of Environmental Vibration

**Figure 44.**
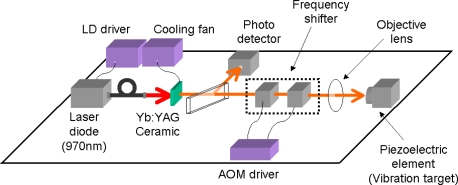
Experimental setup of the self-mixing thin-slice Yb:YAG vibrometry under an environmental vibrations.

Let me show the result of real-time vibrometry performed by the present Yb:YAG ceramic laser possessing a pronounced optical sensitivity in large-amplitude environmental vibrations expected in the actual measurement. The experimental setup is shown in Figure 44. Two PbMoO_4_ acousto-optical modulators (AOM; Hoya-Schott A-150, 80 MHz central frequency) were inserted between the laser and a target to provide a carrier frequency of 2 MHz (*i.e.*, light frequency shift after the round trip). The output beam from the frequency shifter was impinged on a mirror mounted on a piezoelectric element through a microscope objective lens of NA = 0.25 with a focal length of 22.17 mm and a diameter of beam focus estimated to be about 36 mm. Here, we placed an LD driver, a temperature controller (thermoelectric cooler) with air cooling fans, and the vibrating mirror mount (target) on the same table to deliberately produce large-amplitude environmental vibrations, which might be expected in actual measurement circumstances.

Because the optical sensitivity was extremely high in the present Yb:YAG ceramic laser, the carrier-to-noise ratio (CNR) at 2 MHz, in the absence of vibrations of the mirror, easily reached over 50 dB under weak optical feedback. When CNR was increased beyond 60 dB, however, the laser exhibited chaotic relaxation oscillations. Therefore, the intensity feedback ratio was controlled by defocusing the access beam onto the vibrating mirror about 1–2 mm from the focus point of the objective lens.

**Figure 45.**
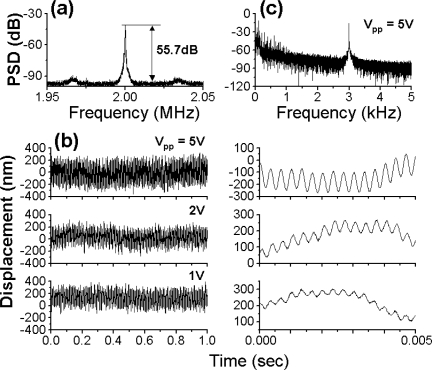
**(a)** Power spectrum around the carrier frequency of the laser output in the absence of vibrations of the mirror. **(b)** Vibration waveforms with decreasing applied peak-to-peak voltage (V_pp_ = 5, 2, and 1V) at a vibration frequency of 3 kHz. The left side shows long-term vibration waveforms indicating environmental vibrations and the right side shows magnified views indicating nanometer-scale vibrations of the piezoelectric element. **(c)** Power spectrum corresponding to Figure 45(b) indicating a sharp peak at 3 kHz and low-frequency environmental vibration components (reprinted with permission from [95]; © 2009, The Japan Society of Applied Physics).

**Figure 46.**
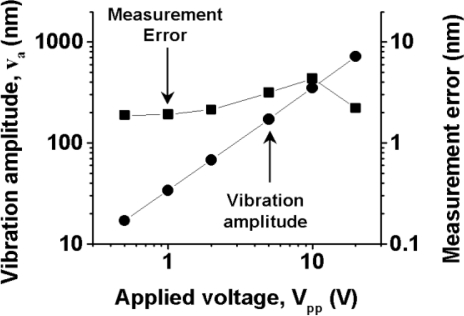
Relationship between the peak-to-peak voltage applied to the piezoelectric element and the measured peak-to-peak vibration amplitude. Measurement errors, *i.e.*, standard deviations, are also shown for each plot (reprinted with permission from [95]; © 2009, The Japan Society of Applied Physics).

The measured power spectrum in the absence of vibrations of the mirror is shown in Figure 45(a), yielding CNR = 56 dB. Note that the power spectrum is broader than expected from the short-term frequency stability of 10 Hz of the AOM drivers we used, because of the presence of FM sidebands resulting from low frequency environmental vibrations. From the comparison between the experimental and numerical simulation results, the feedback ratio was estimated to be −123 dB in the present experiment if we neglect the effect of environmental vibrations. In the case of Nd:GdVO_4_, CNR was decreased to 41 dB. Examples of vibration waveforms detected by the software-defined radio with different voltages applied to the piezoelectric element are shown in Figure 45(b). We can clearly observe nanometer-scale vibrations embedded in low-frequency and large-amplitude environmental vibrations of the instruments as shown in the power spectrum in Figure 45(c), which corresponds to the results shown in Figure 46(b).

The relationship between the voltage applied to the piezoelectric element and the measured vibration amplitude is shown in Figure 46. A linear relationship, which parallels the result measured using the capacitive vibration sensor, is obtained. The lower bound of measurable peak-to-peak vibration amplitude under the present feedback conditions was *v*_a_ = 20 nm in the presence of large-amplitude environmental vibrations.

## 8. Concluding Remark and Outlook

The dynamic effect of highly-sensitive self-mixing intensity modulation inherent in thin-slice solid-state lasers subjected to optical feedback, which was explored by the present author in 1979 [4,5], and several distinct applications to real-time optical metrology systems with extremely high optical sensitivity have been reviewed, focusing mainly on measurements performed in the past 10 years in Tokai University.

The futuristic prospect of laser-diode-pumped, self-mixing thin-slice solid-state metrology could include:

1. Fiber-optic sensing network systems of distant targets based on self-mixing thin-slice solid-state lasers toward high-grade security, environmental metrology, *etc*.
2. Self-mixing lasers coupled to a fiber-optic nanometer probe for high-spatial-resolution analysis of dynamic behaviors of specimens toward cell biology, surface science, *etc*.
3. Highly-sensitive optical detection systems of a single nanometer particle toward molecular biology, efficient contamination analysis, *etc*.
4. Noninvasive plasma diagnostics and scattering measurement of moving electrons.
5. Self-mixing laser topography systems with high spatial resolutions toward medical applications.
6. Self-mixing thin-slice solid-state lasers possessing extreme optical sensitivity with controlled quantum noise toward optical metrology below quantum limit.
7. Self-mixing thin-slice solid-state lasers coupled to a variety of external interferometers for measuring slow/tiny changes in physical values, e.g., displacement, strain, fiber-optic gyroscope, gravitational telescope, *etc*.

Demonstrations of significant ‘killer applications’ of thin-slice self-mixing solid-state laser metrology, which can never be performed by any state-of-the-art technology, are strongly anticipated.

## Figures and Tables

**Table 1.** Measured velocity vector and maximum speed of water flow within the glass pipe. The values expected from our settings for the glass pipe on the optical bench and the syringe pump are also shown for comparison.

	**Velocity (mm/s)**	**θ (degree)**	**ψ (degree)**

**Experimental result**	72.56	9.78	7.00
**Experimental setup**	76.32	10.00	7.25

**Table 2.** Parameters related to the optical sensitivity in self-mixing measurements.

	**3 at.% doped Crystalline 1-mm-thick Nd:GdVO_4_**	**20 at.% doped Crystalline 2-mm-thick Yb:YAG**	**5 at.% doped Ceramic 2-mm-thick Yb:YAG**

**τ (μs)**	90	1100	900
**τ_p_ (μs)**	233	101	176
***K* (10^5^)**	8.86	119	51.1

## Appendix: Singular Value Decomposition (SDV) Analysis of Mixed Samples

The power spectrum density *P*(*ω*) is the linear combination of Lorentzian functions for *N* kind of particles with different sizes. The *i*-th frequency component is:

(A1) $$P(\omega_{i})=\sum_{j=1}^{N}w_{j}\frac{\Gamma_{j}}{{\left(\omega_{i}-{2\omega }_{\mathrm{AOM}}\right)}^{2}+\Gamma_{j}^{2}}$$

where *w_j_* is the weighted parameters and Γ*_j_* is the decay rate of the *j*-th kind of particle and Γ*_j_* = *k*^2^*D*. For *M* frequency components, their relationship can be presented in a matrix form:

$$\left[\begin{array}{c} P(\omega_{1})\\ P(\omega_{2})\\ \vdots \\ P(\omega_{M}) \end{array}\right]=\left[\begin{array}{cccc} \frac{\Gamma_{1}}{{\left(\omega_{1}-{2\omega }_{\mathrm{AOM}}\right)}^{2}+\Gamma_{1}^{2}} & \frac{\Gamma_{2}}{{\left(\omega_{1}-{2\omega }_{\mathrm{AOM}}\right)}^{2}+\Gamma_{2}^{2}} & \cdots & \frac{\Gamma_{N}}{{\left(\omega_{1}{-2\omega }_{\mathrm{AOM}}\right)}^{2}+\Gamma_{N}^{2}}\\ \frac{\Gamma_{1}}{{\left(\omega_{2}-2\omega_{\mathrm{AOM}}\right)}^{2}+\Gamma_{1}^{2}} & \frac{\Gamma_{2}^{2}}{{\left(\omega_{2}-2\omega_{\mathrm{AOM}}\right)}^{2}+\Gamma_{2}^{2}} & \cdots & \frac{\Gamma_{N}^{2}}{{\left(\omega_{2}-2\omega_{\mathrm{AOM}}\right)}^{2}+\Gamma_{N}^{2}}\\ \vdots & \vdots & \vdots & \vdots \\ \frac{\Gamma_{1}}{{\left(\omega_{M}-{2\omega }_{\mathrm{AOM}}\right)}^{2}+\Gamma_{1}^{2}} & \frac{\Gamma_{2}^{2}}{{\left(\omega_{M}-{2\omega }_{\mathrm{AOM}}\right)}^{2}+\Gamma_{2}^{2}} & \cdots & \frac{\Gamma_{N}^{2}}{{\left(\omega_{M}-{2\omega }_{\mathrm{AOM}}\right)}^{2}+\Gamma_{N}^{2}} \end{array}\right]\;\left[\begin{array}{c} w_{1}\\ w_{2}\\ \vdots \\ w_{N} \end{array}\right].$$

We define the power spectrum density matrix **P**, the Lorentzian function matrix **C**, and the weighted parameter matrix **W**. The equation (A1) can read as **P** = **CW**. By using the SVD method, the Lorentzian function matrix **C** can be decomposed as **C** = **UΛV***^T^*, where **U** and **V** are made up of the column vectors *u_i_* and *v_i_* which are the eigenvectors of **CC***^T^* and **C***^T^***C**, respectively. **Λ** is the diagonal matrix of singular values in a descending order *λ*_1_ > *λ*_2_ > … > *λ_N_*. The weighted parameter matrix **W** = **VΛ**^−1^**U***^T^* **P**, *i.e.*,

(A2) $$W=\sum_{i=1}^{N}\frac{1}{\lambda_{i}}(u_{i}^{T}P)v_{i}$$

For brevity, we assume the decay rates Γ_1_, Γ_2_,…,Γ*_M_* to obtain the Lorentzian function matrix **C**. Then, solve the parameters *w*_1_, *w*_2_,…, *w_M_* by SVD method. However, the smaller the values of *λ_i_* the greater is the uncertainty in the solution of **W** exerted by a noise distortion in **P**, because the singular values occur in the denominator in Equation (R2). To avoid the risk of an unstable solution, we truncated the summation in Equation (R2) at *i* = ℓ for $\frac{\lambda_{i}}{\lambda_{1}}>10^{-3}$, $W=\sum_{i=1}^{\ell }\frac{1}{\lambda_{i}}(u_{i}^{T}P)v_{i}$, in the present analysis of the size histogram shown in Figure 23.
